# Sodium mediated deprotonative borylation of arenes using sterically demanding B(CH_2_SiMe_3_)_3_: unlocking polybasic behaviour and competing lateral borane sodiation†

**DOI:** 10.1039/d3sc01705b

**Published:** 2023-05-05

**Authors:** Andreu Tortajada, Leonie J. Bole, Manting Mu, Martin Stanford, Marconi N. Peñas-Defrutos, Max García-Melchor, Eva Hevia

**Affiliations:** a Department für Chemie und Biochemie, Universität Bern Freiestrasse 3 3012 Bern Switzerland eva.hevia@unibe.ch; b School of Chemistry, CRANN and AMBER Research Centres, Trinity College Dublin College Green Dublin 2 Ireland garciamm@tcd.ie; c IU CINQUIMA/Química Inorgánica, Facultad de Ciencias, Universidad de Valladolid 47071-Valladolid Spain

## Abstract

The deprotonative metalation of organic molecules has become a convenient route to prepare functionalised aromatic substrates. Amongst the different metallating reagents available, sodium bases have recently emerged as a more sustainable and powerful alternative to their lithium analogues. Here we report the study of the sterically demanding electrophilic trap B(CH_2_SiMe_3_)_3_ for the deprotonative borylation of arenes using NaTMP (TMP = 2,2,6,6-tetramethylpiperidide) in combination with tridentate Lewis donor PMDETA (PMDETA = *N*,*N*,*N*′,*N*′′,*N*′′-pentamethyldiethylenetriamine). Using anisole and benzene as model substrates, unexpected polybasic behaviour has been uncovered, which enables the formal borylation of two equivalents of the relevant arene. The combination of X-ray crystallographic and NMR monitoring studies with DFT calculations has revealed that while the first B–C bond forming process takes place *via* a sodiation/borylation sequence to furnish [(PMDETA)NaB(Ar)(CH_2_SiMe_3_)_3_] species, the second borylation step is facilitated by the formation of a borata-alkene intermediate, without the need of an external base. For non-activated benzene, it has also been found that under stoichimetric conditions the lateral sodiation of B(CH_2_SiMe_3_)_3_ becomes a competitive reaction pathway furnishing a novel borata-alkene complex. Showing a clear alkali-metal effect, the use of the sodium base is key to access this reactivity, while the metalation/borylation of the amine donor PMDETA is observed instead when LiTMP is used.

## Abstract 2

Mechanistic studies on sodium-mediated borylation of arenes, uncover a unique polybasic behaviour for the formal borylation of two equivalents of the relevant arene as well as the competing formation of a borata-alkene complexes.

## Graphical abstract

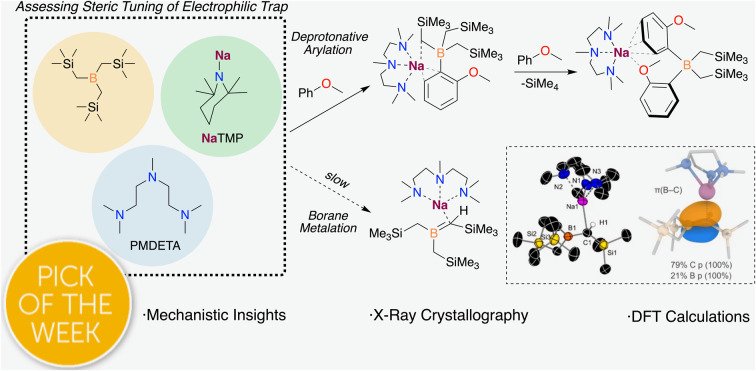

## Introduction

Organic boranes and boronates have become very powerful building blocks in organic synthesis, being fundamental in key transformations such as Suzuki–Miyaura cross-couplings,^1^ Chan-Lan couplings,^2^ homologation reactions *via* 1,2-metalate rearrangements^3^ or radical-mediated reactions.^4^ One of the traditional doorways to these organoboron reagents has been the reaction of organometallic nucleophiles (typically organolithium or Grignard reagents) with boron-based electrophiles, such as BX_3_ (X = F, Cl),^5^ B(OR)_3_ ^6^ or HBpin.^7^ However, the use of organometallic reagents presents some drawbacks, namely they are prepared from pre-functionalised molecules (usually organic halides), their formation is often troublesome, and they show a low functional group tolerance. To overcome some of these limitations, the use of strong non-nucleophilic bases in deprotonative metalation has emerged as a powerful alternative. These allow the metalated species to be generated from unfunctionalized molecules at low temperatures and/or in low concentrations, which can then be reacted with electrophilic boron molecules to prepare the corresponding boron derivatives.^8^ However, most studies reported to date lack an evaluation of the substituent's influence in the boron-based moieties.

**Fig. 1:**
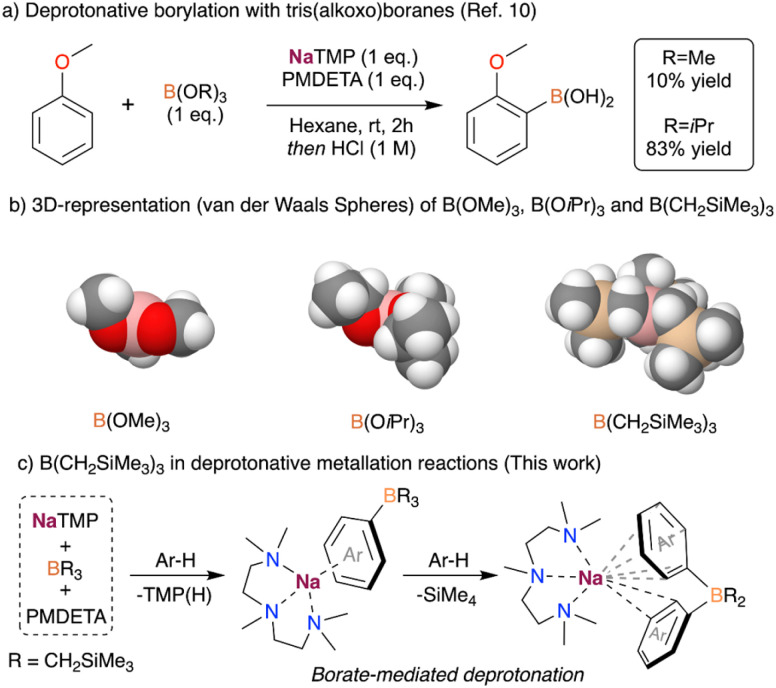
Trivalent boron species as trapping agents in sodium-mediated deprotonative metalation reactions.

Recently, we have shown that the use of NaTMP (TMP = 2,2,6,6-tetramethylpiperidide) in the deprotonation of arenes results in an equilibrium between the metalated arene and the sodiated amide, which was key to develop a catalytic hydrogen isotope exchange for the deuteration of arenes.^9^ The use of the bulky Lewis acidic B(O*i*Pr)_3_ was essential to push the equilibrium forward, allowing the deprotonative borylation of a wide range of aromatic compounds with good yields. This reaction enabled the functionalization of arenes ranging from reactive fluoroarenes to simple and much less reactive aromatic substrates such as benzene, naphthalene or anisole.^10^ During the study of this transformation, we realised that the use of B(OMe)_3_ instead of B(O*i*Pr)_3_ almost suppressed the formation of the desired C–B bond (Fig. 1a). NMR reaction monitoring studies and the trapping of key reaction intermediates suggest that this particular reactivity is underpinned by the steric incompatibility of NaTMP and B(O*i*Pr)_3,_ while the tridentate amine PMDETA (PMDETA = *N*,*N*,*N*′,*N*′′,*N*′′-pentamethyldiethylenetriamine) induces the de-aggregation of NaTMP, enhancing its kinetic basicity.

Intrigued by these findings, we wondered whether the use of other bulkier boranes (Fig. 1b) could improve the base-mediated borylation of arenes. Previously, in collaboration with Mulvey, we successfully employed the lithium amide LiTMP in combination with a sterically demanding group 13 metal trap, such as Al(TMP)*i*Bu_2_ and Ga(CH_2_SiMe_3_)_3_, for the regioselective metalation of fluoroarenes and diazines.^11^ Key for the success of these Trans-Metal-Trapping (TMT) approaches is the lack of co-complexation between the two homometallic components.^12^ Inspired by this work, the bulky trialkyl borane B(CH_2_SiMe_3_)_3_ was chosen as a boron-based electrophile for this study. This reagent has been reported to have a reduced Lewis acidity when compared to its heavier analogues Al_2_(CH_2_SiMe_3_)_6_ and Ga(CH_2_SiMe_3_)_3_.^13^ This, coupled with the smaller size of B compared to Al and Ga, makes B(CH_2_SiMe_3_)_3_ a great candidate for deprotonative metalations in combination with NaTMP, minimising the opportunities for co-complexation with the sodium amide.

Assessing the influence of increasing steric incompatibility in sodium-mediated borylation reactions, here we report the reactivity of NaTMP/PMDETA/B(CH_2_SiMe_3_)_3_ combinations with arenes. Using anisole and benzene as model substrates, we uncover a unique polybasic behaviour which enables the formal borylation of two equivalents of the relevant arene (Fig. 1c). By combining spectroscopic studies of the trapped key reaction intermediates and density functional theory (DFT) calculations, we provide mechanistic insights into this unexpected reactivity. Furthermore, we report the sodiation of the trialkylborane as a competitive reaction pathway when using non-activated substrates such as benzene under stochiometric conditions.

## Results and discussion

**Fig. 2:**
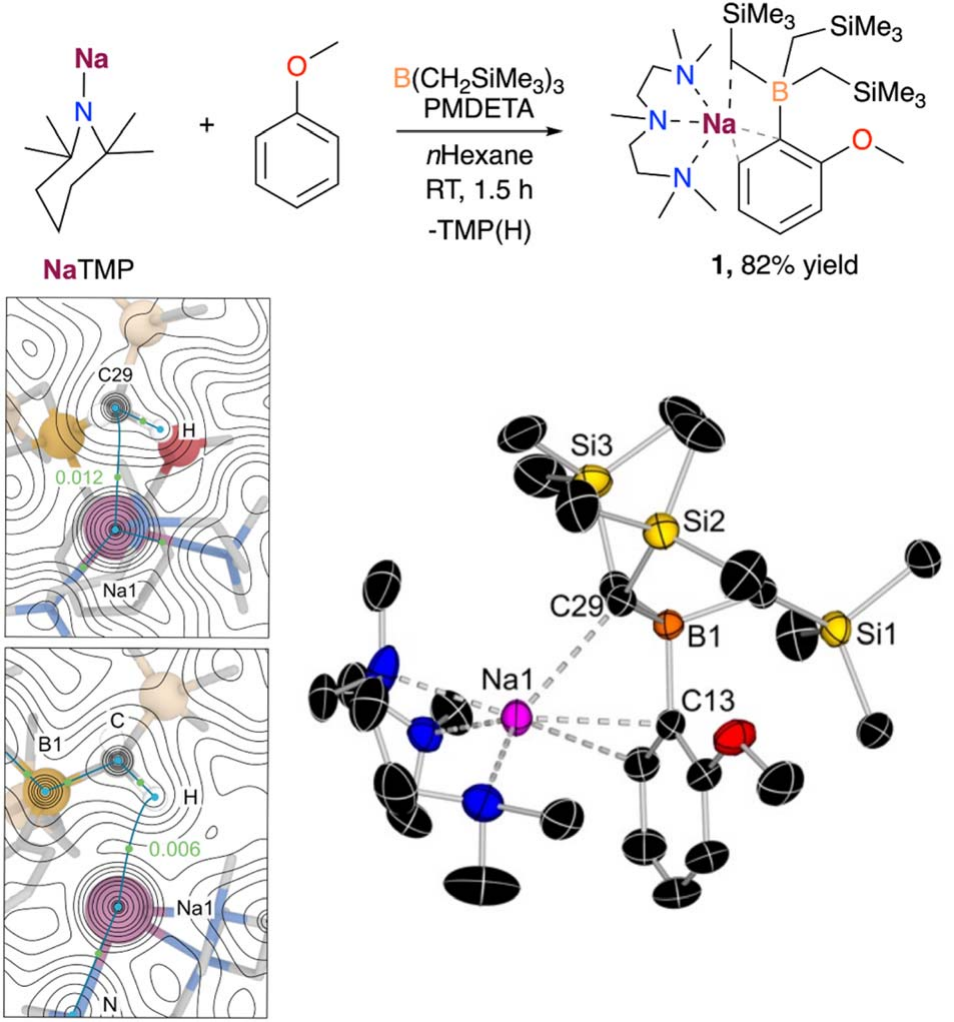
Deprotonative borylation of anisole carried out in this work. Bottom left: Electron density contour maps for complex 1 generated from the QTAIM analysis depicting the computed bond critical points (BCPs) and nuclear critical points (NCPs) as green and blue dots, respectively. The *ρ*(*r*) values at the selected BCPs for (Na1⋯H) and (Na1⋯C29) are shown in a.u. Bottom right: X-ray crystal structure of [(PMDETA)NaB(C_6_H_4_–OMe)(CH_2_SiMe_3_)_3_] (1) with 50% probability displacement ellipsoids. H atoms have been omitted for clarity.

We began our studies using anisole, a classical substrate in directed ortho metalation chemistry.^14^ Using an equimolecular mixture of NaTMP and PMDETA as a metalating base in combination with B(CH_2_SiMe_3_)_3_, the sodium borate [(PMDETA)NaB(C_6_H_4_–OMe)(CH_2_SiMe_3_)_3_] (1) could be obtained at room temperature after 1.5 h in 82% yield (Fig. 2). Mechanistic DFT studies (see ESI† for details) revealed that the monomeric PMDETA·NaTMP is the active species in the deprotonation of anisole, yielding a sodiated intermediate which subsequently undergoes C–B bond formation to afford complex 1 (see Fig. S1†). According to calculations, the most energy demanding step is the deprotonation of anisole with a Gibbs energy barrier in *n*-hexane of +16.1 kcal mol^−1^, while C–B bond formation requires a lower barrier of +14.3 kcal mol^−1^. Interestingly, a close inspection of TS1 (Fig. S2 in ESI†) revealed that contrasting with previous mechanistic studies on the directed-ortho-lithiation of anisole, where it has been proposed that the substrate interacts with the RLi reagent by forming a Li–O bond in a prelithiation complex as a reaction intermediate (Complex Induced Proximity Effect, CIPE),^15^ here Na adopts a perpendicular disposition, π-engaging with the C that is going to experience the metalation, and does not interact with the OMe substituent.

Overall, the formation of 1 is predicted to be both kinetically and thermodynamically feasible at room temperature, in agreement with experimental observations. This reactivity, however, contrasts with that previously reported when using NaTMP/TMEDA in combination with Ga(CH_2_SiMe_3_)_3_, which results in the relevant ortho-gallated product in a modest 17% yield.^11*b*^ This has been attributed to the formation of a sodium gallate *via* co-complexation of the two homometallic components, which lacks sufficient basicity to promote the C–H bond cleavage in anisole. The undesired co-complexation is suppressed in this work by the smaller-sized B atom and the weaker Lewis acidity of B(CH_2_SiMe_3_)_3_.

The selective formation of the C–B bond was confirmed by X-ray crystallographic studies, with compound 1 displaying an interacting ion-pair structure wherein the Na atom solvated by the tridentate donor PMDETA is forming a π-interaction with the aromatic ring *via* two C atoms (Na–C distances of 2.6381(13) and 2.7985(15) Å, Fig. 2). Surprisingly, the oxygen atom does not present a coordinative interaction with the sodium centre, presumably due to the more favourable interactions between sodium and the aromatic ring in anisole and the B(CH_2_SiMe_3_)_3_ moiety. Further examination of the structure of 1*via* quantum theory of atoms and molecules (QTAIM) analysis confirmed the presence of a non-covalent interaction between Na and one of the CH_2_ moieties of the CH_2_SiMe_3_ group in close proximity to Na. This is evidenced by the presence of bond critical points (BCP) between Na1⋯H and Na1⋯C29, with electron density of 0.006 and 0.012 a.u. (Fig. 2), and the DFT-calculated distances of 2.427 and 3.081 Å, respectively, which agrees with the observed Na1⋯C29 distance of 2.8247(13) Å in the solid state. While this latter distance is longer than those reported for isolated [(L)NaCH_2_SiMe_3_]_n_ complexes (from *ca.* 2.467 to 2.732 Å),^16^ it is closer to those found in related heterobimetallic systems such as [(TMEDA)Na(TMP)M(CH_2_SiMe_3_)_2_] (M = Zn, Fe), where the alkyl group is bridging the Na and other divalent metals (Na–C distances of 2.787 and 2.838 Å for NaZn and NaFe systems, respectively).^17^

**Fig. 3:**
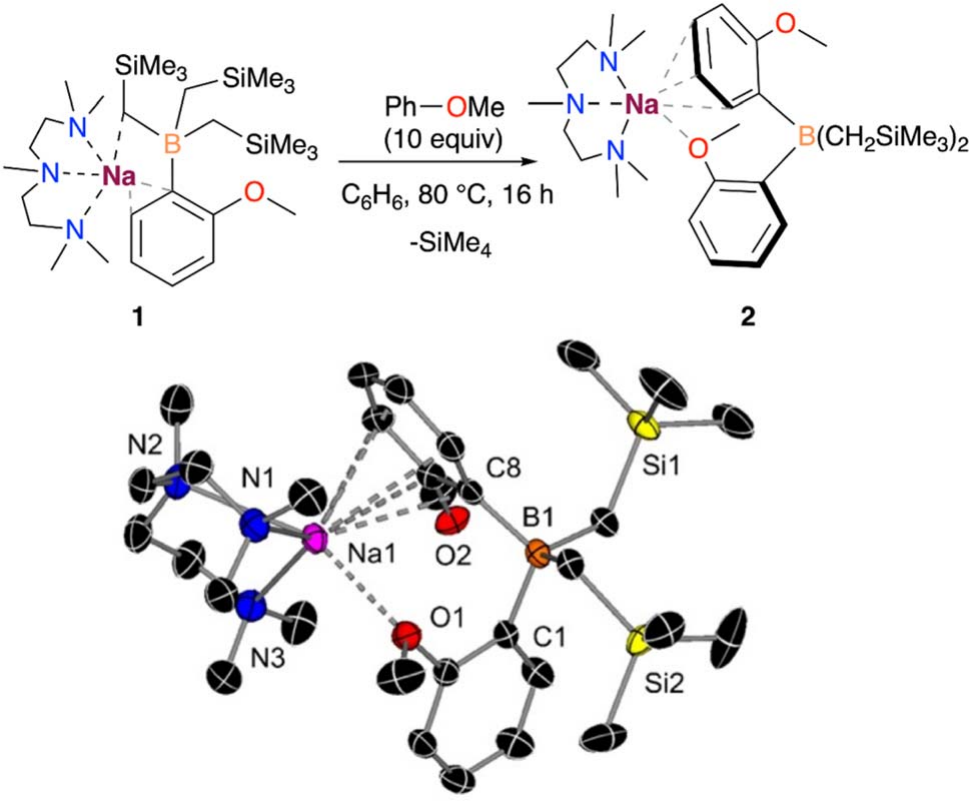
Deprotonative borylation of anisole with 1. X-ray crystal structure of (PMDETA)Na[(C_6_H_4_–OMe)_2_B(CH_2_SiMe_3_)_2_] (2) with 50% probability displacement ellipsoids. H atoms have been omitted for clarity.

While monitoring the formation of 1 in solution *via*^1^H NMR spectroscopy, a small amount of a second product was observed in the presence of a slight excess of anisole, containing the characteristic resonances for a 2-anisyl fragment. Interestingly, this second product was formed exclusively when 1 was reacted with 10 equivalents of anisole in benzene at 80 °C, obtaining the borate [(PMDETA)NaB(C_6_H_4_–OMe)_2_(CH_2_SiMe_3_)_2_] (2) with the full conversion of compound 1, based on the recorded ^1^H NMR data. The crystallization of the crude reaction mixture at low temperatures allowed the isolation of 2 in 41% yield (Fig. 3).

X-ray crystallographic studies confirmed the interacting ion-pair structure of the sodium borate 2 in which boron is bound to two –CH_2_SiMe_3_ groups and two C2-anisyl fragments. In this structure the {Na(PMDETA)}^+^ cation binds to the two aryls, although in two distinct coordination modes. Particularly, one of them π-engages with the Na fragment in an *η*^6^ fashion (Na–C distances ranging from 2.9543(17) to 3.1403(18) Å), whereas the second ring interacts through a Na–O dative bond *via* the OMe substituent (Na–O distance of 2.4549(13) Å).

**Fig. 4:**
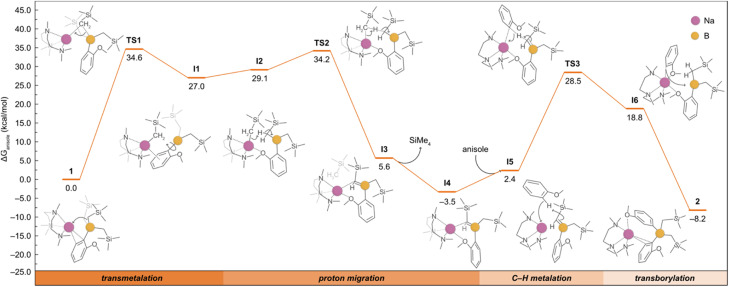
Gibbs energy profile for the deprotonative borylation of anisole from 1 to afford 2. Gibbs energies are calculated at the experimental conditions of 353.15 K and 1 atm, in anisole as solvent (see ESI† for details). The bottom bar highlights the four main reaction steps, and the detailed mechanism is shown with the aid of curly arrows.

Although the formation of 2 could be rationalised with 1 undergoing a ligand redistribution process, the fact that 2 forms quantitatively when using an excess of anisole rules out this possibility. Furthermore, when monitoring the reaction by ^1^H NMR, the formation of SiMe_4_ was also observed, consistent with the formal borylation of a second equivalent of anisole employing one of the alkyl groups present in 1. We also found that further heating of 2 in the presence of an excess of anisole does not promote the activation of any further alkyl groups on the borate. In addition, we noted that using B(O*i*Pr)_3_ instead of B(CH_2_SiMe_3_)_3_ under the same conditions does not lead to the borylation of a second equivalent of anisole. Intrigued by these findings, which suggest that one of the *a priori* inert alkyl groups at the boron undergoes activation towards the deprotonative borylation of a second equivalent of anisole, we next investigated the reaction mechanism for the formation of 2 by DFT calculations. Based on previous experimental studies,^16*b*^ we began by considering the monometallic sodium complex [(PMDETA)NaCH_2_SiMe_3_] as the active species. However, the formation of [(PMDETA)NaCH_2_SiMe_3_] alongside with [B(C_6_H_4_–OMe)(CH_2_SiMe)_2_] from 1 is predicted to be thermodynamically very unfavourable (*i.e.*, +25.2 kcal mol^−1^). This energy difference increases the overall barriers of the located mono- and dimeric homometallic Na transition states to +43.1 and +41.6 kcal mol^−1^ (see Fig. S4 and S5†), respectively, rendering these pathways kinetically unfeasible under reaction conditions of this work. In addition, we explored other monometallic Na species, but all of them exhibited energy barriers higher than *ca.* +45 kcal mol^−1^ (Fig. S3†). These results were further supported *via* a control experiment which showed no reactivity when [(PMDETA)NaCH_2_SiMe_3_] and compound 1 were mixed in a C_6_D_6_ solution. Next, we considered the deprotonation of the substrate with the –CH_2_SiMe_3_ group from the NaB interacting ion-pair 1, whereby the H from the substrate is directly transferred to the C atom of the alkyl group, breaking the C–B bond and forming a Na–C bond and SiMe_4_. Again, all the transition states located for this process (Fig. S3†) feature energy barriers above +38.7 kcal mol^−1^, making this reaction unlikely at the experimental conditions. We then posited that the high barriers may be due to the cleavage of the strong B–C bond and proposed the alternative mechanism, depicted in Fig. 4, which involves the intramolecular release of SiMe_4_ with the concomitant formation of a B=C bond prior to the C–H deprotonation of anisole. This pathway begins with the intramolecular transmetalation of one of the CH_2_SiMe_3_ groups in 1, giving rise to the first reaction intermediate I1. This process occurs *via*TS1 and requires an energy barrier of +34.6 kcal mol^−1^, which seems reasonable under the experimental conditions, *i.e.* 80 °C, excess of substrate, 12 h. From I1, there is a rearrangement to form I2, followed by the migration of the H from the borylated CH_2_ fragment to the sodiated -CH_2_SiMe_3_ group *via* a transition state (TS2) with an overall barrier of +34.2 kcal mol^−1^. Subsequently, the elimination of SiMe_4_ to yield the sodium borata-alkene I4 occurs in an exergonic process by −3.5 kcal mol^−1^, driving the reaction forward. Once formed, the intermediate I4 is able to deprotonate the second equivalent of anisole thanks to the nucleophilic carbon in the C=B bond, leading to I6 in which the newly generated C2-anisyl fragment is bound to Na. This step takes place *via*TS3 with an overall energy barrier of 32.0 kcal mol^−1^. The final product 2 is then obtained *via* intramolecular transborylation, a step which makes the whole process exergonic by −8.2 kcal mol^−1^.

**Fig. 5:**
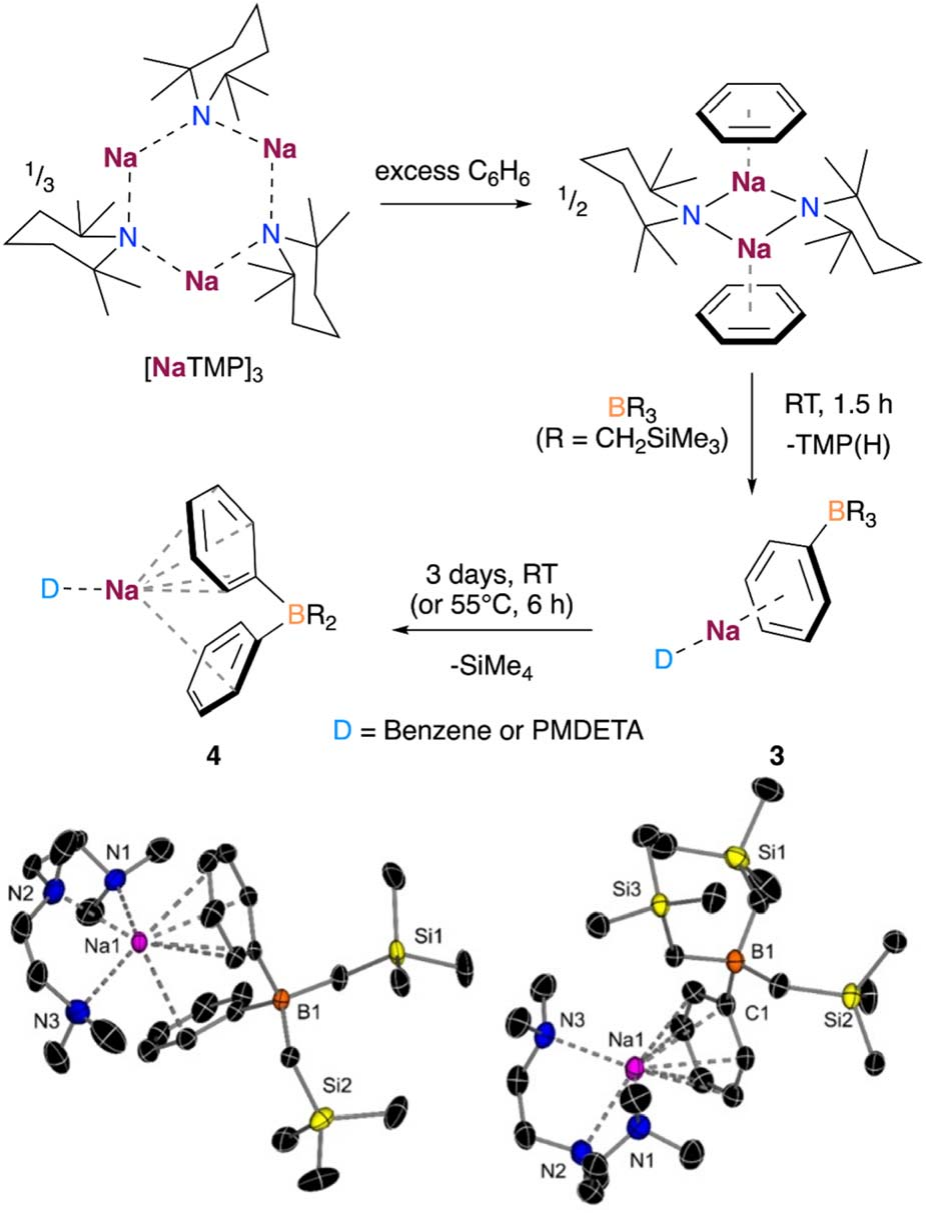
Deprotonative borylation of benzene. X-ray crystal structure of (PMDETA)Na[PhB(CH_2_SiMe_3_)_3_] (3, right) and (PMDETA)Na[Ph_2_B(CH_2_SiMe_3_)_2_] (4, left) with 50% probability displacement ellipsoids. H atoms have been omitted for clarity.

Based on the above findings and the ability of NaTMP to promote the sodiation of unactivated arenes such as benzene,^9,10^ although only negligible yields in the absence of a trapping agent, we decided to investigate the deprotonative borylation of benzene using B(CH_2_SiMe_3_)_3_. When benzene was used as a solvent, we observed by ^1^H DOSY NMR the formation of a solvated dimer of the sodium amide, as shown in Fig. 5. De-aggregation of the NaTMP trimer increases the basicity of the sodium amide, enabling the formation of PhNa which can be trapped *in situ* by the reaction with B(CH_2_SiMe_3_)_3_ to form the corresponding borate [(benzene)_*x*_NaB(Ph)(CH_2_SiMe_3_)_3_]. Addition of the chelating donor PMDETA allowed the crystallization and isolation of the borate [(PMDETA)NaB(Ph)(CH_2_SiMe_3_)_3_] (3) in 32% crystalline yield.

Interestingly, in a similar way to 1, when the reaction mixture was reacted in the presence of an excess of benzene for longer periods of time or at higher temperatures, the deprotonative borylation of a second equivalent of benzene was observed. This produced the borate [(benzene)_*x*_NaB(Ph)_2_(CH_2_SiMe_3_)_2_], which could be crystalized in the presence of PMDETA as [(PMDETA)NaB(Ph)_2_(CH_2_SiMe_3_)_2_] (4) in 60% yield. Both borates 3 and 4 present a contacted ion-pair structure, wherein the Na atom is coordinated to a benzene ring in a *η*^6^ fashion and one PMDETA ligand. In addition, the second benzene ring in 4 features a single contact with the Na atom through one of the *ortho* C atoms of the ring (Fig. 5). This second deprotonation/arylation demonstrates that the borate mediated metalation of arenes is not restricted to anisole and can occur as well with benzene if present in vast excess (as solvent). Similar to anisole, monitoring the reaction by ^1^H NMR spectroscopy revealed the formation of SiMe_4_, suggesting a similar reaction mechanism for the second borylation of benzene.

Since benzene is both the substrate and solvent of the reaction and is present in a vast excess, we next attempted the borylation reaction under stoichiometric conditions, like with anisole in the formation of 1 (Fig. 2). Hence, we reacted one equivalent of benzene with an equimolar mixture of NaTMP and PMDETA in *n*-hexane. ^1^H NMR monitoring studies revealed that this reaction does not result in the sodium borate 3, but a novel borata-alkene [(PMDETA)Na(Me_3_Si)HC=B(CH_2_SiMe_3_)_2_] (5). This complex displays a characteristic signal in the ^11^B NMR spectrum with a chemical shift of 49.3 ppm, which is an intermediate value between tricoordinate B(CH_2_SiMe_3_)_3_ (*δ* = 78.9 ppm) and tetracoordinated borates (ranging from −15 to −13 ppm), and similar to other reported sodium borata-alkenes (40.9–42.2 ppm).^18*a*^

Compound 5 is formed through the competing deprotonation of the boron trap B(CH_2_SiMe_3_)_3_ by NaTMP·PMDETA and could be rationally isolated as a pure crystalline solid in 59% yield when the reaction was carried out in *n*-hexane and in the absence of benzene (see ESI† for details). While previous studies have shown the ability of organolithium reagents to deprotonate alkylboranes without reacting with the Lewis acid boron centre to form the corresponding borata-alkene,^19^ the use of other heavier alkali-metal amides for the selective deprotonation of these compounds remains scarce.

**Fig. 6:**
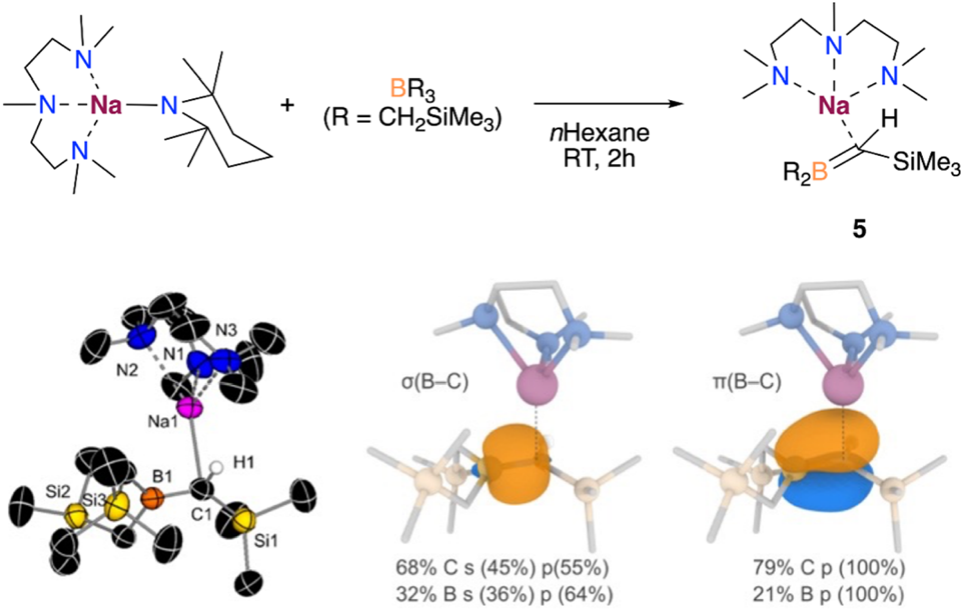
Top: Deprotonative metalation of B(CH_2_SiMe_3_)_3_. Bottom left: X-ray crystal structure of [(PMDETA)Na(Me_3_Si)HC=B(CH_2_SiMe_3_)_2_] (5) with 50% probability displacement ellipsoids. H atoms, except the one bound to C1, have been omitted for clarity. Bottom middle and right: isosurfaces (isovalue = 0.06 a.u.) depicting the *σ*(B–C) and π(B–C) natural bond orbitals (NBOs) involved in the formation of the C=B double-bond in 5. The different contributions from the atomic orbitals to the NBOs are also shown.

The crystal structure of 5 was confirmed by X-ray diffraction, which displays a shortened C–B bond (1.476(3) *vs.* 1.609(3) and 1.610(3) Å, Fig. 6). This distance is similar to that found in C=B double bonds, which typically range from *ca.* 1.377 to 1.570 Å.^18^ The nature of the C–B bond in 5 was further investigated by natural bonding orbital (NBO) analysis, which confirmed the double bond character based on the occupancies of the relevant σ and π-bond NBOs (1.97 and 1.87 a.u., respectively, see Fig. 6). The higher contribution observed from the C compared to B in the NBOs also highlights the nucleophilic character of the C atom, which accounts for the observed reactivity, as discussed below. The multiple bond character was also confirmed through QTAIM analysis by examining the electron density at the C–B BCPs (0.195 a.u., see Table S3 and Fig. S5† for details).

Reactivity studies showed that 5 is unreactive towards benzene or anisole metalation, ruling out this species as an intermediate in the formation of the sodium borates 1 and 3. The different reactivity observed for 5 and the previously suggested I4 (Fig. 4) might arise from the higher steric bulk of the two –CH_2_SiMe_3_ groups, stabilising the borata-alkene 5 and allowing its isolation. In addition, 5 is not observed when reacting NaTMP/PMDETA and B(CH_2_SiMe_3_)_3_ with an equimolar amount of anisole (*vide supra*). Thus, these findings point out the formation of 5 as a deactivating pathway in the deprotonative borylation of non-activated arenes such as benzene, which can be minimised by using a large excess of substrate.

**Fig. 7:**
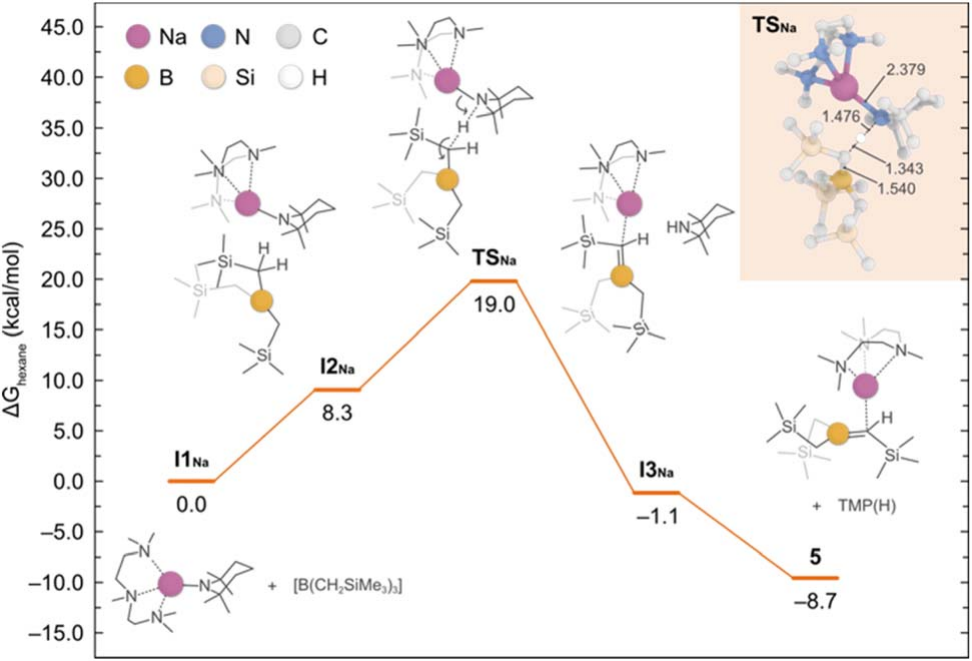
Gibbs energy profile for the deprotonative sodiation of B(CH_2_SiMe_3_)_3_ with NaTMP·PMDETA to afford 5. Gibbs energies are calculated at the experimental conditions of 298.15 K and 1 atm, in *n*-hexane as solvent. The detailed mechanism is shown with the aid of curly arrows. In the inset shows the optimised structure of TS_Na_ with the most relevant bond distances (in Å).

To shed light into the formation of compound 5, we performed DFT calculations to elucidate the underlying reaction mechanism. Based on these results, we propose that PMDETA promotes the de-aggregation of the starting reactant {NaTMP}_3_ to yield the more activated monomeric species [(PMDETA)Na(TMP)]. The formation of such species is predicted to be exergonic by −12.3 kcal mol^−1^ and in equilibrium with the dimeric form [{(PMDETA)Na(TMP)}_2_] (−0.2 kcal mol^−1^). However, because of the steric hindrance between [(PMDETA)Na(TMP)] and B(CH_2_SiMe_3_)_3_, the co-complexation reaction is precluded and the sodiation of the borylated CH_2_ group is favoured instead. This process, depicted in Fig. 7, involves a monomeric transition state (TSNa) with an energy barrier of 19.0 kcal mol^−1^; the dimeric equivalent was also considered and found to be higher in energy of 30.6 kcal mol^−1^. This relatively low barrier of 19.0 kcal mol^−1^ and the fact that the overall reaction is exergonic by −8.7 kcal mol^−1^ is consistent with the experimental observation of product 5 at room temperature.

Given that 5 can be accessed *via* direct sodiation of the borane B(CH_2_SiMe_3_)_3_ and the nucleophilic character of the B=C bond, we posited that the borata-alkene I4 could be an en route intermediate to yield the bis(aryl) sodium borates 2 and 4 (Fig. 4). Unfortunately, all the attempts to isolate I4 from the reaction mixture in the presence of an excess of anisole were unsuccessful. This is, however, in line with DFT calculations which predict that intermediate I4 may not accumulate in solution since the rate of its disappearance (TS3, 32.0 kcal mol^−1^) is faster than its formation (TS1, 34.6 kcal mol^−1^). Moreover, I4 is thermodynamically less stable than 2 (by 4.7 kcal mol^−1^), shifting the overall reaction forward towards the formation of 2. Although I4 could not be isolated experimentally, further evidence supporting the formation of this intermediate species was found when 1 was heated in cyclohexane-*d*_12_ at 80 °C in the absence of anisole, observing partial decomposition with multiple products and concomitant formation of SiMe_4_.

**Scheme 1:**
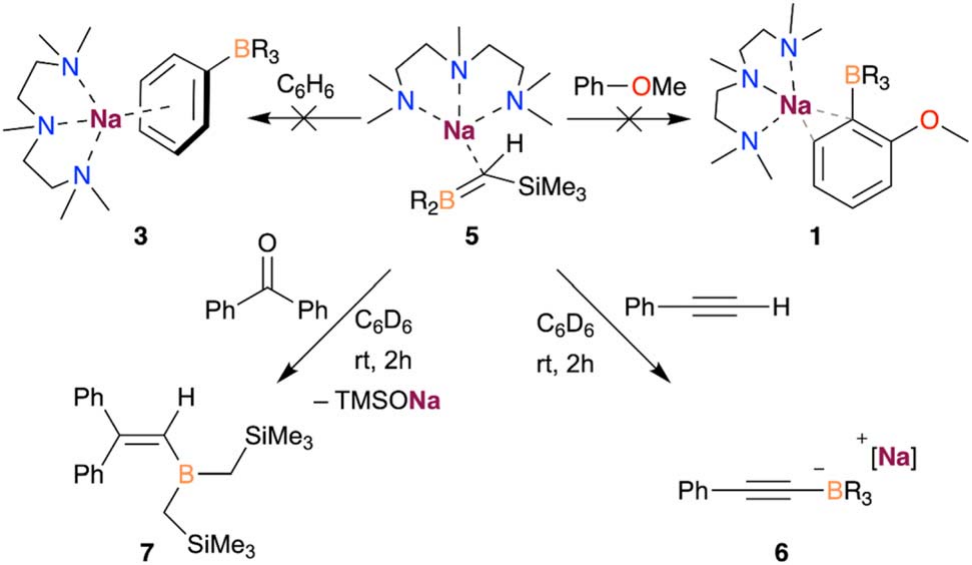
Reactivity of compound 5 with benzophenone and phenylacetylene.

Further reactivity studies with compound 5, summarised in Scheme 1, show that this species is sufficiently basic to deprotonate the terminal proton of phenylacetylene, forming the corresponding borate [(PMDETA)NaB(C_6_H_5_C≡C)(CH_2_SiMe_3_)_3_] (6), as evidenced by ^1^H and ^11^B NMR spectroscopies (see ESI† for details). Moreover, 5 can react with benzophenone *via* nucleophilic attack, leading to the sodium alkoxide which subsequently undergoes Peterson elimination to afford the corresponding vinyl borane 7.^20^

**Fig. 8:**
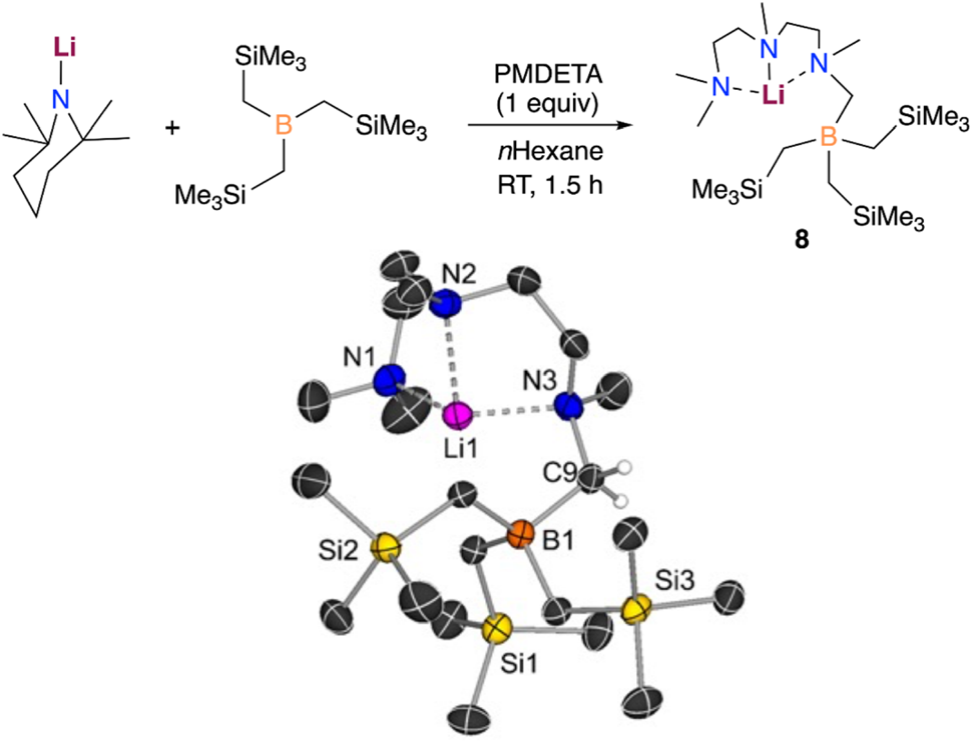
Deprotonative borylation of the Lewis donor PMDETA with LiTMP/B(CH_2_SiMe_3_)_3_. X-ray crystal structure of [(PMDETA^−^)LiB(CH_2_SiMe_3_)_3_] (8) with 30% probability displacement ellipsoids. H atoms, except the ones attached to C9, have been omitted for clarity.

Evidencing a clear alkali-metal effect, we noted that LiTMP/PMDETA fails to metalate B(CH_2_SiMe_3_)_3_. Interestingly, an equimolar mixture of these components furnished the borylation product [({Me_2_NCH_2_CH_2_N(Me)CH_2_CH_2_N(Me)CH_2_})LiB(CH_2_SiMe_3_)_3_] (8), which could be isolated as crystals in a 39% yield (Fig. 8).

In this case, the lithium amide promotes the regioselective deprotonative borylation of one of the methyl groups of the Lewis donor PMDETA, leaving the alkyl groups on boron untouched. This is well supported by our computational results which show that the formation of the analogue lithiation product (5-Li) requires a higher energy barrier (+28.4 kcal mol^−1^*vs.* +19.0 kcal mol^−1^). In addition, the formation of 5-Li is predicted to be thermodynamically less favourable than that of the experimentally observed borylation product 8 by +4.1 kcal mol^−1^ (see Fig. S6†). Notably, the stability of the compounds 5 and 8 with Na is reversed (5 is more stable by −3.6 kcal mol^−1^). Hence, only the reactivity of LiTMP/PMDETA and B(CH_2_SiMe_3_)_3_ is reminiscent of what we previously reported when reacting MTMP (M = Li, Na), PMDETA and Ga(CH_2_SiMe_3_)_3_ in *n*-hexane.^11*b*^

## Conclusions

The electrophilic trapping of *in situ* generated sodiated arenes is a powerful strategy for the synthesis of aryl borates, although the nature of the Lewis acidic boron molecules has been typically overlooked. This study of the bulky B(CH_2_SiMe_3_)_3_ as electrophilic trap has allowed the synthesis and full characterisation of borates 1 and 3, resulting from the enhancement of the metalating ability of the sodium amide NaTMP in combination with the donor PMDETA and the borane trapping agent. Using an excess of substrate and higher temperatures, these complexes can promote a second deprotonative borylation in the absence of an additional base to furnish the bis(aryl) borates 2 and 4. DFT calculations and NMR monitoring studies have allowed us to propose a reaction mechanism for this transformation, in which the initial migration of the alkyl group to sodium promotes deprotonation leading to a borata-alkene intermediate which engages in the deprotonation of a second equivalent of arene substrate. The structurally related borata-alkene 5 was prepared *via* the deprotonation of B(CH_2_SiMe_3_)_3_ and could be structurally characterized by X-ray diffraction. Key mechanistic experiments and DFT calculations support that this borata-alkene intermediate does not participate in the formation of borates 1 and 3, but it can be reacted with more activated molecules such as phenylacetylene or benzophenone, accessing new trialkylborane derivatives. Finally, when LiTMP was used instead of NaTMP, the metalation of the Lewis donor PMDETA was observed without formation of the borata-alkene product, showing a clear alkali-metal effect.

Altogether, the findings reported in this work provide a deeper understanding of the role of each of the components and their close interplay in the deprotonative borylation of arenes, while shedding light on the constitution of the organometallic intermediates involved and the importance of the choice of alkali-metal.

## Data availability

CCDC codes 2245605–2245610 contain the crystallographic data for this article and can be accessed at: https://www.ccdc.cam.ac.uk. All the DFT data underlying this work, including the Cartesian coordinates of the modelled species, are openly accesible *via* the following ioChem-BD online dataset: http://dx.doi.org/10.19061/iochem-bd-6-226.

## Author contributions

M. Stanford, L. J. Bole and A. Tortajada performed the experimental work. M. Mu and M. N. Peñas-Defrutos carried out the computational studies. M. García-Melchor and E. Hevia supervised the work. All authors participated in the writing of the manuscript and approved its last version.

## Conflicts of interest

There are no conflicts to declare.

## Supplementary Material

SC-014-D3SC01705B-s001

SC-014-D3SC01705B-s002
