# Supplementary material for: Sodium mediated deprotonative borylation of arenes using sterically demanding B(CH2SiMe3)3: unlocking polybasic behaviour and competing lateral borane sodiation
Source: Chem Sci. 2023 May 5;14(24):6538–45. doi: 10.1039/d3sc01705b (PMC10283504; doi:10.1039/d3sc01705b)
Supplement: SC-014-D3SC01705B-s001 [file SC-014-D3SC01705B-s001.pdf]

Electronic Supplementary Material (ESI) for Chemical Science.
This journal is © The Royal Society of Chemistry 2023

Supplementary Information for:

Sodium Mediated Deprotonative Borylation of Arenes Using Sterically Demanding $B(CH_2SiMe_3)_3$: Unlocking Polybasic Behaviour and Competing Lateral Borane Sodiation

Andreu Tortajada,[a] Leonie J. Bole,[a‡] Manting Mu,[b‡] Martin Stanford,[a] Marconi N. Peñas-Defrutos,[b,c] Max García-Melchor,[b*] and Eva Hevia[a*]

[a] Department für Chemie und Biochemie, Universität Bern, Freiestrasse 3, 3012 Bern, Switzerland

[b] School of Chemistry, CRANN and AMBER Research Centres, Trinity College Dublin, College Green, Dublin 2, Ireland

[c] IU CINQUIMA/Química Inorgánica, Facultad de Ciencias, Universidad de Valladolid, 47071-Valladolid, Spain.

Contents

General Methods 3

Synthetic Procedures 4

- Preparation of $B(CH_2SiMe_3)_3$ 4
- Preparation of **1** 4
- Preparation of **2** 6
- Preparation of **3** 8
- Preparation of **4** 11
- Preparation of **5** 13
- Preparation of **8** 15
- Reactivity with compound **5** 17
- Control experiments with compound **1** 22

X-Ray Crystallographic Details 24

Computational Methods 28

Gibbs energy profile for the Formation of Complex 1 29

Transition State Energies for the Formation of 2 from 1 30

Quantum Theory of Atoms in Molecules (QTAIM) Analysis 31

Mechanism for the hypothetical deprotonative lithiation of $B(CH_2SiMe_3)_3$ with LiTMP·PMDETA 32

Cartesian coordinates and energies of the modelled structures 33

References 34

General Methods

All procedures were conducted using standard Schlenk line and glove box techniques under an inert atmosphere of argon. Hexane was degassed, purified and collected via an MBraun SPS 5 and stored over 4 Å molecular sieves for at least 24 hours prior to use. THF was dried by heating to reflux over sodium-wire/benzophenone ketyl radical and stored over 4 Å molecular sieves for 24 hours prior to use. Deuterated solvents (C_6D_6 and D_8-THF) were purchased from VWR, dried over NaK alloy for 16 hours and then cycled through three rounds of degassing by employing a freeze-pump-thaw method. The deuterated solvents were then collected via vacuum transfer and stored argon atmosphere over 4 Å molecular sieves.

All reagents were purchased from Sigma Aldrich, Fluorochem, VWR or Acros Organics. PMDETA (*N,N,N',N'',N''*-pentamethyldiethylenetriamine) and TMEDA (*N,N,N′,N′*-tetramethylethylenediamine), 2,2,6,6-tetramethylpiperidine and anisole were purified by drying them over CaH_2 for 16 hours, before collecting by vacuum distillation. The reagents were stored in an argon-sealed ampule or Schlenk over 4 Å molecular sieves for at least 24 hours prior to use.

NMR spectra were recorded on Bruker spectrometers operating at 300 MHz. ^{1}H NMR spectra: 300.1 MHz, ^{13}C NMR spectra: 75.5 MHz. Spectra were analysed using MestReNova and referenced internally to the corresponding residual protio solvent peaks.

Elemental analyses (C, H and N) were conducted with a Flash 2000 Organic Elemental Analyser (Thermo Scientific). Samples were prepared in the glovebox under argon atmosphere and sealed in an air-tight container prior to analyses. All results were obtained by the Analytical Research and Services Schürch Group of the University of Bern. Samples were weighed on a Mettler Toledo balance with ± 2 μg resolution and sample weights from 1-3 mg were used. For calibration, cysteine was used as a reference material. The presented values are the average of determinations in triplicate to ensure consistency.

Synthetic Procedures

- Preparation of $B(CH_2SiMe_3)_3$

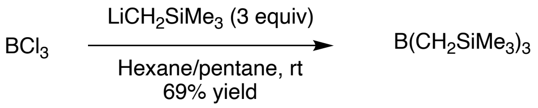

A solution of BCl_3 in hexane (15 mL, 1M, 15 mmol) was added dropwise to a solution of $LiCH_2SiMe_3$ in pentane (45 mL, 1M, 45 mmol) at 0 °C. The resulting white suspension was stirred at room temperature for two hours before filtering through a P4 frit. The solids were washed with hexane (3×10 mL) and the filtrates combined. The volatiles were removed under vacuum to afford the crude product which was purified by distillation under vacuum (100 °C, 0.1 mbar) to afford the pure product as a colourless oil (2.8 g, 69%).

NMR data were in accordance with the literature.[1]

^{1}H NMR (300.1 MHz, C_6D_6, 300 K): δ (ppm) 0.94 (s, 6H, $C\underline{H}_2$ ×3), 0.13 (s, 27H, $SiMe_3$ ×3).

^{11}B NMR (128.4 MHz, C_6D_6, 300 K): δ (ppm) 78.9 (br).

- Preparation of **1**

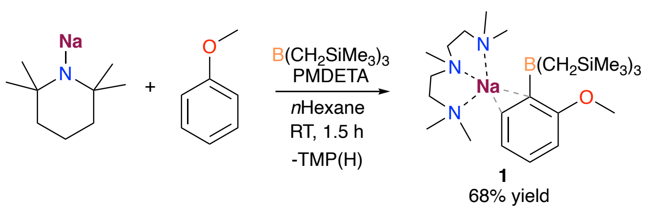

Anisole (108 µL, 108 mg, 1 mmol) was added to a solution of $B(CH_2SiMe_3)_3$ (272 mg, 1 mmol) in hexane (4 mL) at ambient temperature. The solution was stirred for 20 minutes before being added to a suspension of NaTMP (162 mg, 1 mmol) in hexane (6 mL) giving a brown suspension. PMDETA (209 µL, 173 mg, 1 mmol) was then added with no observable changes. The reaction was stirred for a further 45 minutes and then toluene (2 mL) was added to give a brown solution. Crystallisation at –30 °C gave brown crystals which were washed with hexane (5 mL) at –30 °C to give the pure compound (390 mg, 0.68 mmol, 68%) (NMR yield = 82%, using C_6Me_6 as internal standard).

^{1}H NMR (400.1 MHz, C_6D_6, 300 K): δ (ppm) 7.83 (d, 1H, J_{HH} = 6.5 Hz, Ar-$\underline{H}^3$), 7.04–7.00 (m, 1H, Ar-$\underline{H}^5$), 6.92 (td, 1H, J_{HH} = 7.2, 1.1 Hz, Ar-$\underline{H}^4$), 6.58 (d, 1H, 7.9 Hz, Ar-$\underline{H}^6$), 3.58 (s, 3H, O-$C\underline{H}_3$), 1.84 (s, 3H, N-$C\underline{H}_3$), 1.68 (s, 12H, N-$C\underline{H}_3$ ×4), 1.62 (br s, 8H, PMDETA $C\underline{H}_2$ ×4), 0.35 (s, 27H, $SiMe_3$ ×3), 0.02–0.00 (m, 6H, $B(C\underline{H}_2SiMe_3)$ ×3).

^{13}C{^{1}H} NMR (100.6 MHz, C_6D_6, 300 K): δ (ppm) 164.4 (s, Ar $\underline{C}^1$-OMe), 158.0 (m, Ar $\underline{C}^2$-B), 134.1 (s, Ar $\underline{C}^3$), 124.8 (s, Ar $\underline{C}^5$), 120.9 (s, Ar, $\underline{C}^4$), 111.5 (s, Ar, $\underline{C}^6$), 57.0 (s, PMDETA $\underline{C}H_2$ ×2), 55.6 (s, O-$\underline{C}H_3$), 53.6 (s, PMDETA $\underline{C}H_2$ ×2), 45.5 (s, $N(\underline{C}H_3)_2$ ×4), 44.4 (s, $N\underline{C}H_3$), 16.8 (q, J_{CB} = 40 Hz, $\underline{C}H_2SiMe_3$ ×3), 4.0 (s, $SiMe_3$ ×3).

^{11}B{^{1}H} NMR (128.4 MHz, C_6D_6, 300 K): δ (ppm) –13.9.

Elemental Analysis Calculated(%) for $C_{28}H_{63}BN_3NaOSi_3$: C, 58.40; H, 11.03; N, 7.30; Found (%): C, 57.98; H, 10.97; N, 7.13.

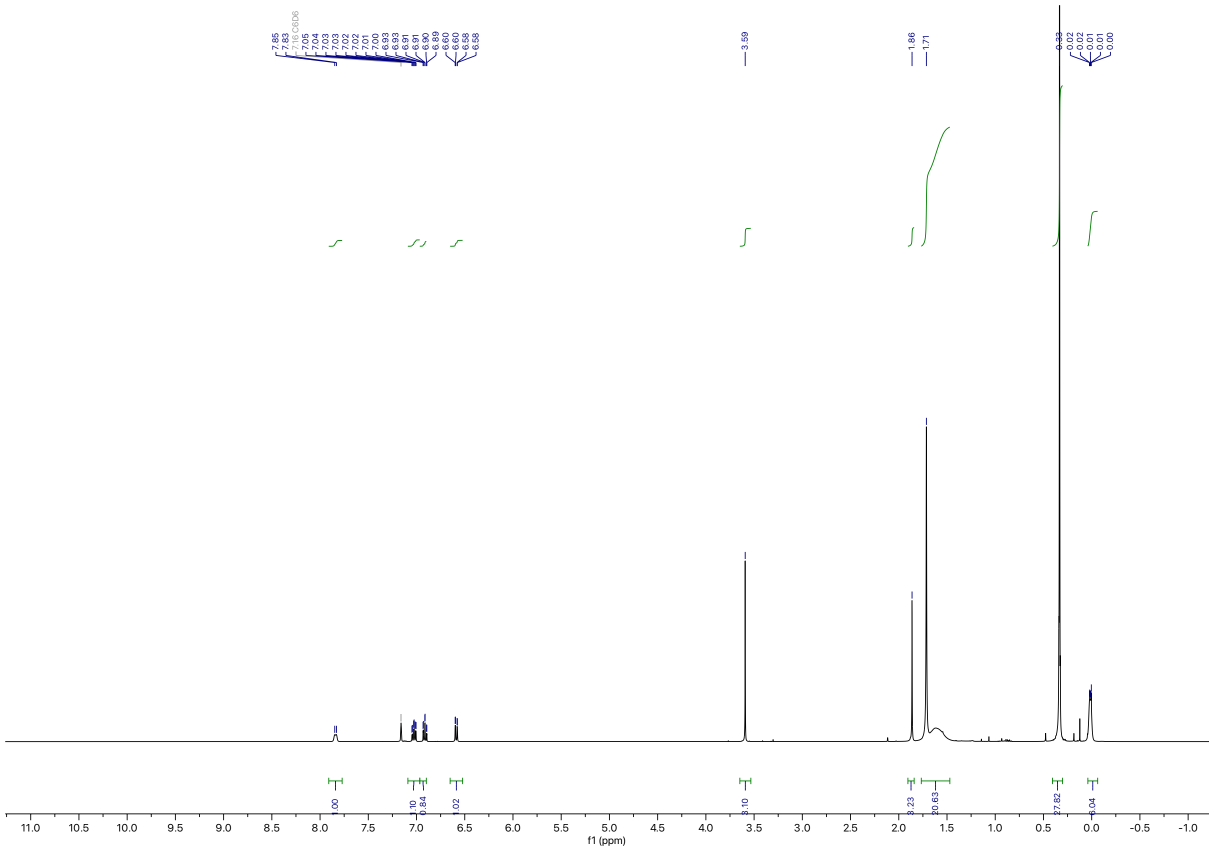

Figure NMR 1. ^{1}H NMR spectrum of compound **1** in C_6D_6.

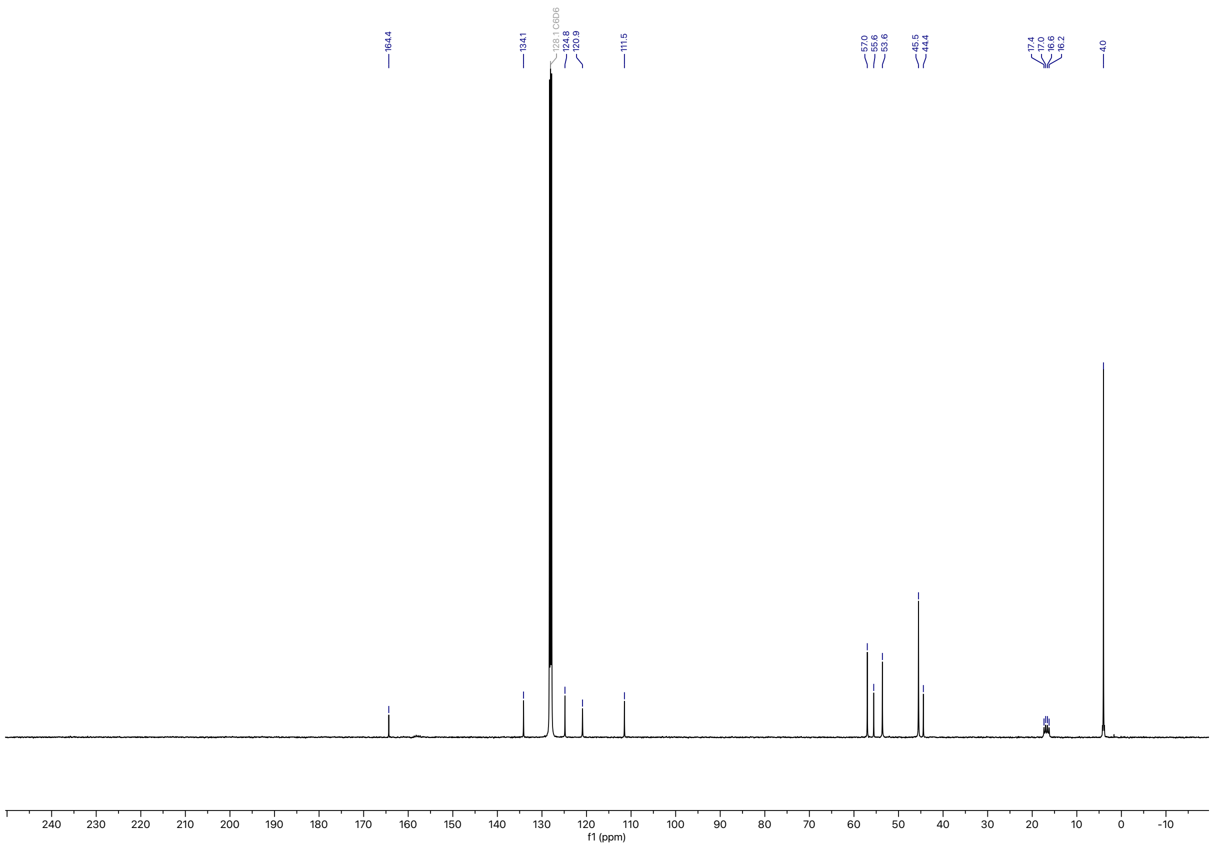

Figure NMR 2. ^{13}C NMR spectrum of compound **1** in C_6D_6.

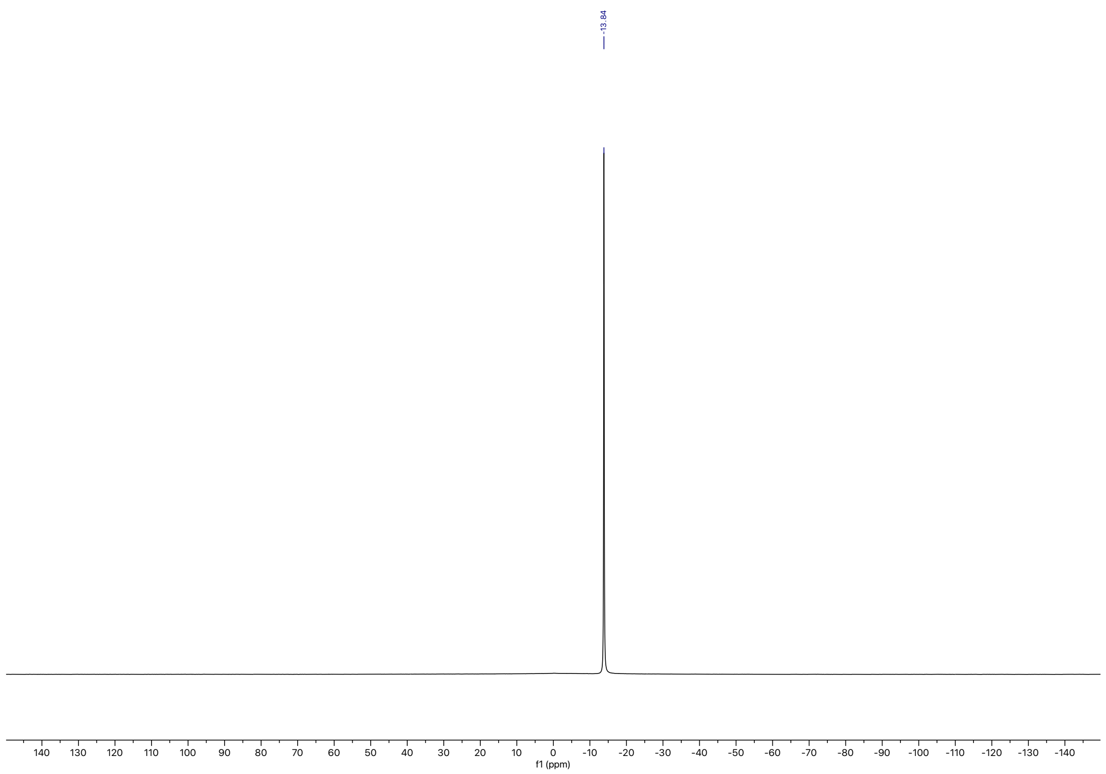

*Figure NMR 3. ^{11}B NMR spectrum of compound **1** in C_6D_6.*

- Preparation of **2**

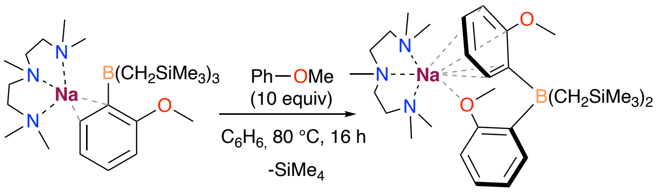

Compound **1** (288.0 mg, 0.5 mmol) was dissolved in benzene (5 mL) and anisole (540 µL, 5 mmol) was added. The reaction mixture was then heated to reflux for 16h and then cooled down to room temperature. Upon cooling, colourless crystals deposited and were isolated by filtration to give compound **4** (122.1 mg, 41% yield). (NMR monitoring of the reaction of **1** with anisole show full conversion to compound **2**).

^{1}H NMR (300 MHz, C_6D_6): δ 8.19 (s, 2H, Ar-$\underline{H}^3$), 7.08 – 6.97 (m, 4H, Ar-$\underline{H}^4$ & Ar-$\underline{H}^5$), 6.58 – 6.47 (m, 2H, Ar-$\underline{H}^6$), 3.43 (s, 6H, O-C$\underline{H}_3$), 1.64 – 1.49 (m, 23H, PMDETA), 0.65 – 0.55 (m, 4H, B-C$\underline{H}_2$SiMe$_3$), 0.23 (s, 18H, SiMe$_3$).
^{13}C{^{1}H} NMR (75 MHz, C_6D_6): δ 163.7 (s, Ar $\underline{C}^1$-OMe), 159.9 – 157.3 (m, Ar $\underline{C}^2$-B), 135.8 (s, Ar $\underline{C}^3$), 124.0 (s, Ar $\underline{C}^5$), 121.7 (s, Ar $\underline{C}^4$), 113.4 (s, Ar, $\underline{C}^6$), 57.9 (s, PMDETA $\underline{C}H_2$ ×2), 57.3 55.6 (s, O-$\underline{C}H_3$), 55.1 (s, PMDETA $\underline{C}H_2$ ×2), 45.3 (s, N($\underline{C}H_3$)$_2$ ×4), 43.9 (s, N$\underline{C}H_3$), 15.4 – 12.1 (m, $\underline{C}H_2$SiMe$_3$), 3.4 (s, SiMe$_3$).

^{11}B{^{1}H} NMR (96 MHz, C_6D_6): δ -13.5.

Elemental Analysis Calculated(%) for $C_{31}H_{59}BN_3NaO_2Si_2$: C, 62.49; H, 9.98; N, 7.05; Found (%): C, 62.27 H, 9.97; N, 7.24.

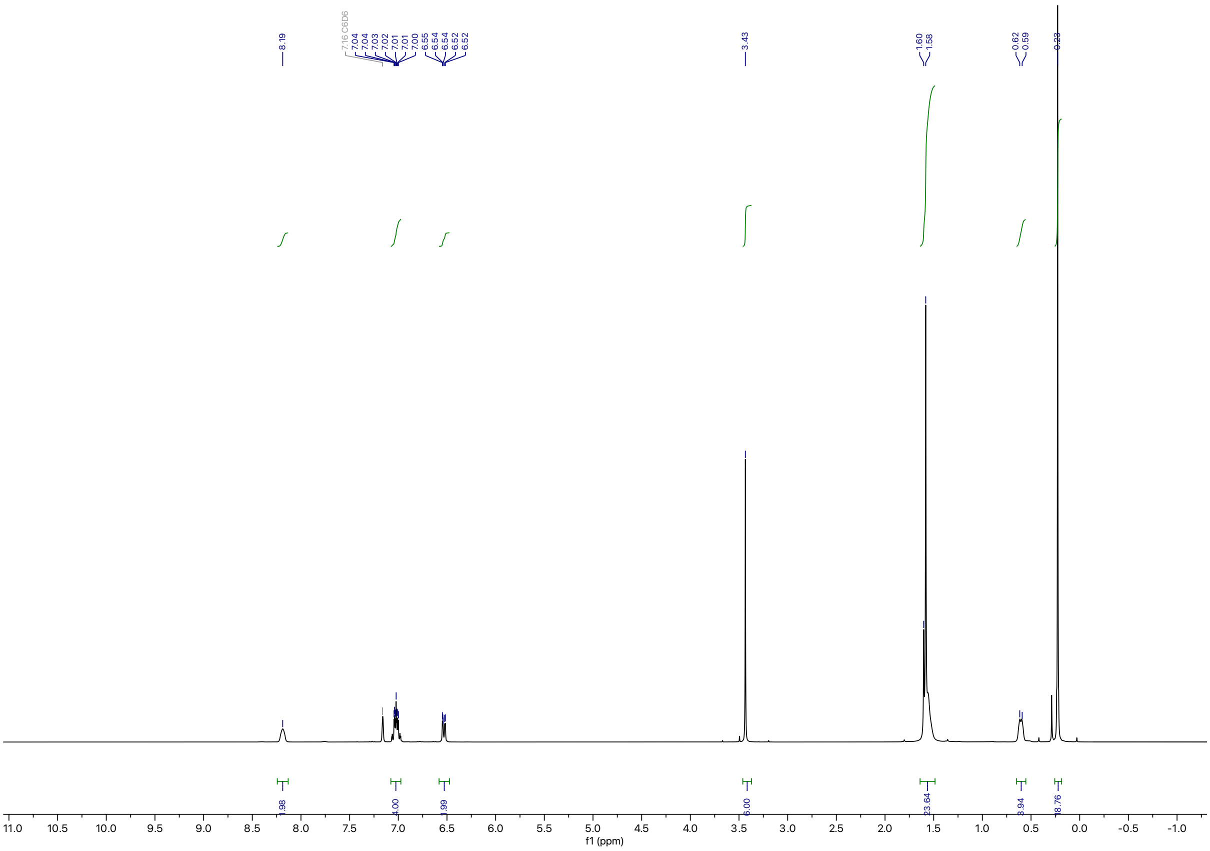

*Figure NMR 4. ^{1}H NMR spectrum of compound **2** in C_6D_6.*

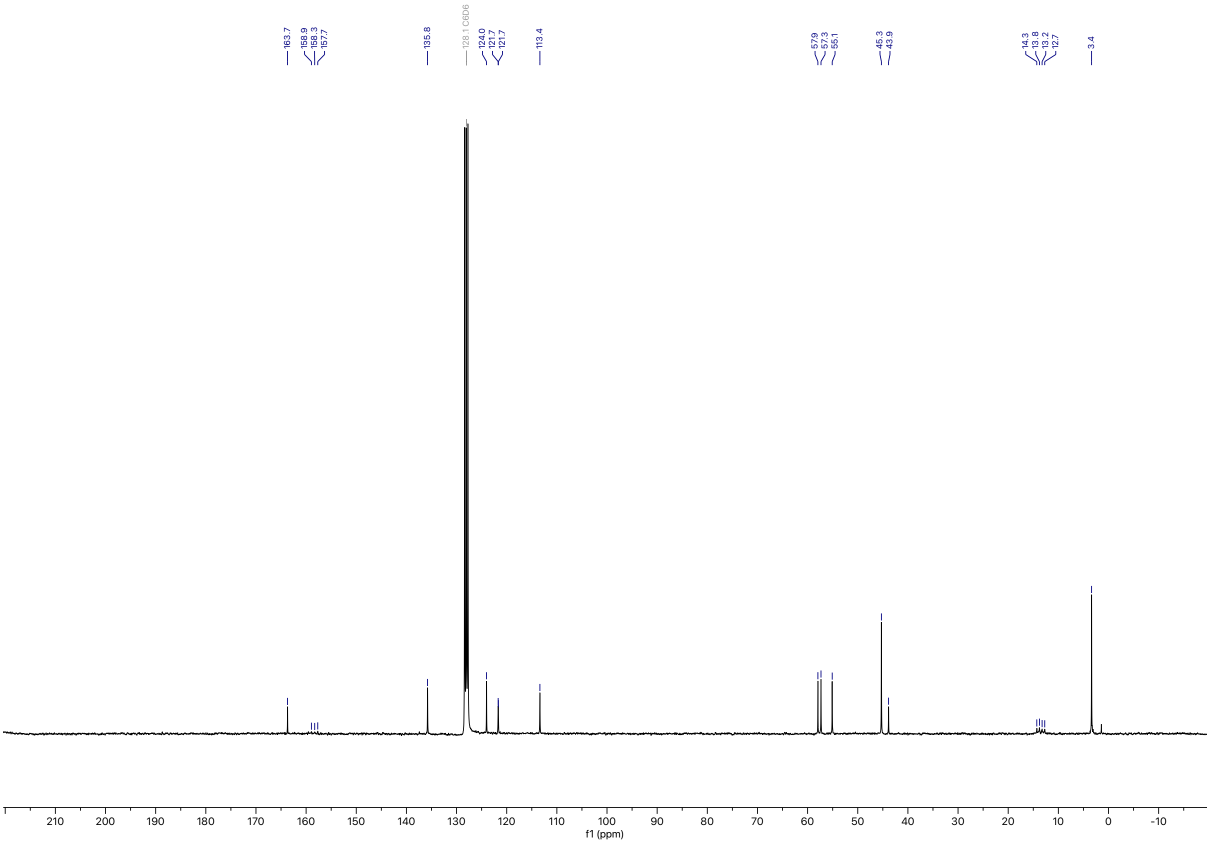

*Figure NMR 5. ^{13}C NMR spectrum of compound **2** in C_6D_6.*

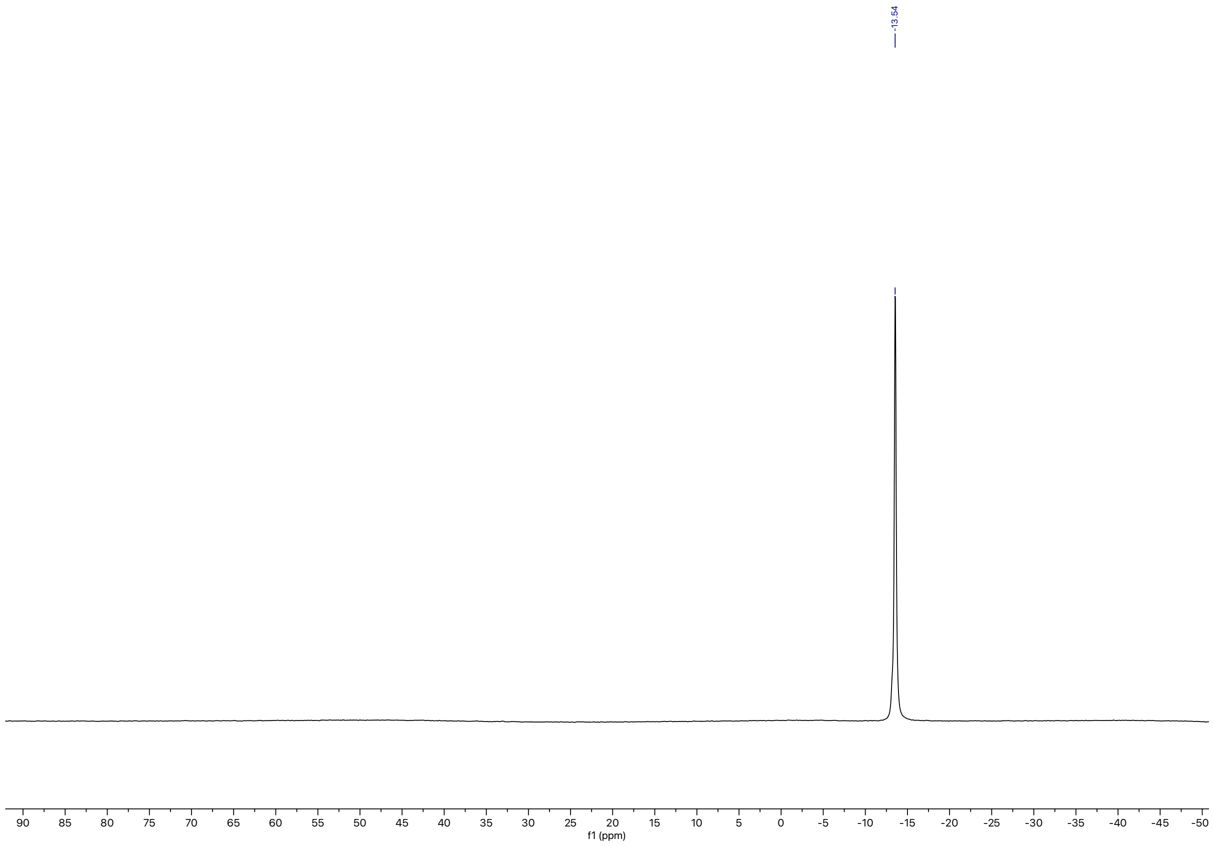

*Figure NMR 6. ^{11}B NMR spectrum of compound **2** in C_6D_6.*

- Preparation of **3**

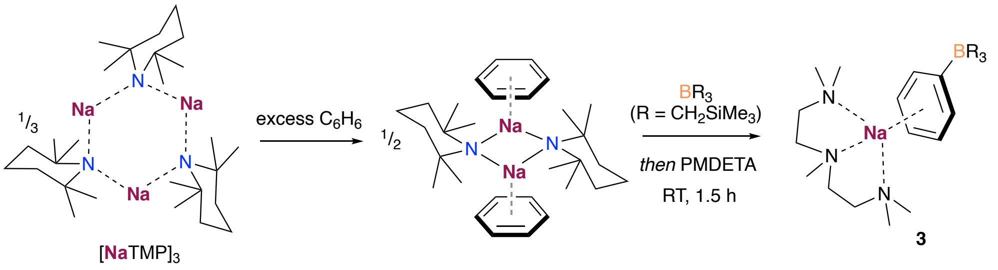

A suspension of $B(CH_2SiMe_3)_3$ (272 mg, 1 mmol) and NaTMP (162 mg, 1 mmol) in benzene (5 mL) was stirred at room temperature for a few minutes. PMDETA (210 μL, 1 mmol) was added giving a bright yellow solution which was stirred for a further 90 minutes. The volatiles were removed *in vacuo* and the resulting oily solid was extracted with hexane (7 mL). The hexane fraction was concentrated by half and cooled to −30 °C to afford colourless crystals of **2** suitable for X-ray diffraction (168, 0.32 mmol, 32%).

^{1}H NMR (400.1 MHz, C_6D_6, 300 K): δ (ppm) 7.87 (br s, 2H, *o*-Ar-H), 7.09 (t, 2H, J_{HH} = 7.2 Hz, *m*-Ar-H), 6.84 (t, 1H, J_{HH} = 7.2 Hz, *p*-Ar-H), 1.70 (s, 3H, PMDETA CH_3 ×1), 1.66 (s, 12H, PMDETA CH_3 ×4), 1.49-1.68 (br m, 8H, PMDETA CH_2 ×4), 0.40 (s, 27H, $SiMe_3$ ×3), 0.02–−0.01 (m, 6H, -$B(CH_2SiMe_3)_3$).

^{13}C{^{1}H} NMR (100.6 MHz, C_6D_6, 300 K): δ (ppm) 174.8 (br s, Ar C-B), 133.4 (s, Ar C_{Ph}-H), 127.0 (s, Ar C_{Ph}-H), 122.6 (s, Ar C_{Ph}-H), 56.9 (s, PMDETA CH_2), 53.6 (s, PMDETA CH_2),

45.6 (s, PMDETA $\underline{C}H_3$), 44.3 (s, PMDETA $\underline{C}H_3$), 19.0–17.8 (m, -B($\underline{C}H_2SiMe_3)_3$), 3.9 (s, $SiMe_3$ ×3).

$^{11}B\{^1H\}$ NMR (128.4 MHz, C_6D_6, 300 K): δ (ppm) −13.42 (s).

Elemental Analysis Calc. for $C_{21}H_{55}BN_3NaSi_3$: C, 59.41; H, 11.26; N, 7.70, found (%): C, 59.10; H, 11.38; N, 7.81.

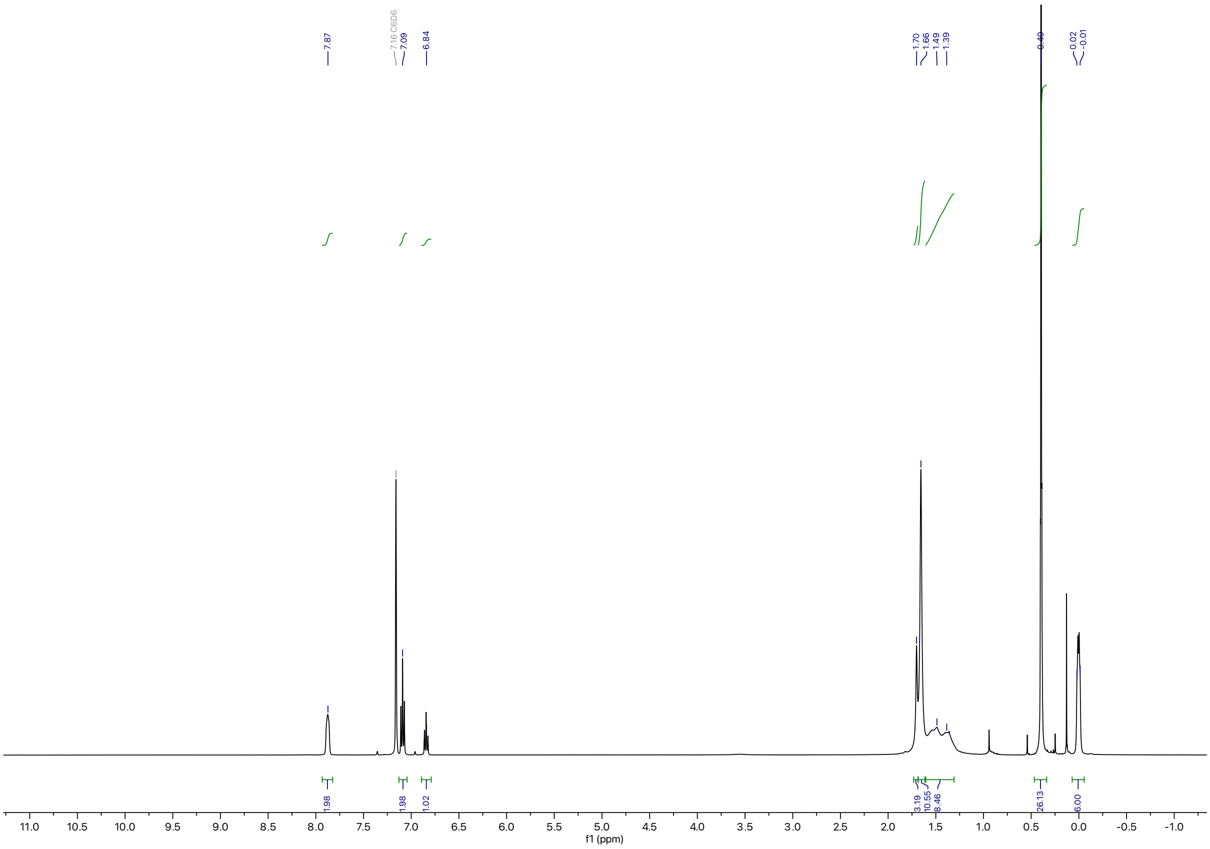

*Figure NMR 7. ^1H NMR spectrum of compound **3** in C_6D_6.*

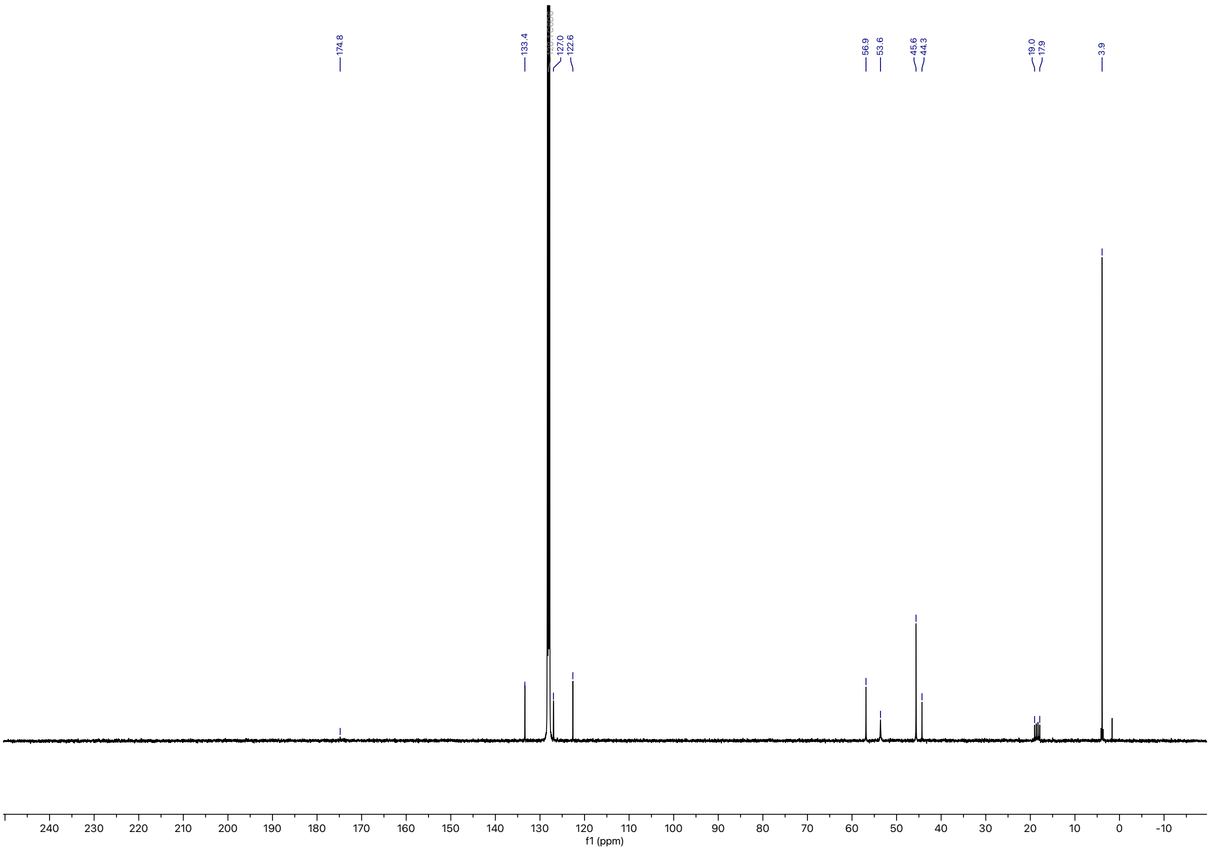

Figure NMR 8. ^{13}C *NMR spectrum of compound* ***3*** *in* C_6D_6.

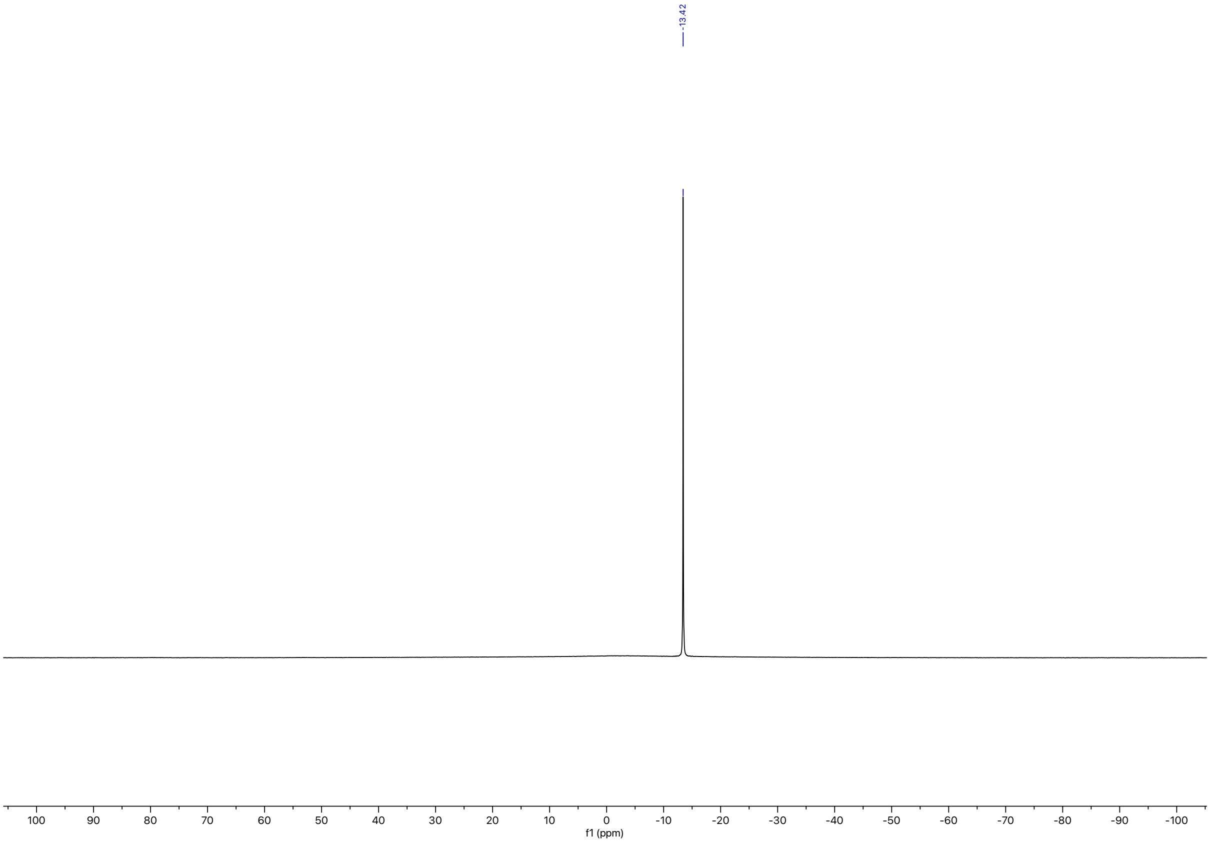

Figure NMR 9. ^{11}B *NMR spectrum of compound* ***3*** *in* C_6D_6.

- Preparation of **4**

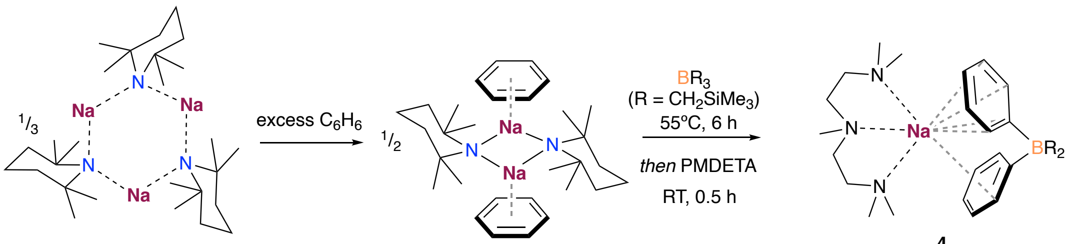

$[NaTMP]_3$ **4**

A suspension of $B(CH_2SiMe_3)_3$ (272 mg, 1 mmol) and NaTMP (162 mg, 1 mmol) in benzene (5 mL) was heated to 55 °C to give a pale brown solution. After heating for six hours, the solution was cooled to ambient temperature and PMDETA (210 µL, 1 mmol) was added which led to a slight yellow colour. The solution was stirred for a further 30 minutes before the volatiles were removed *in vacuo* to afford an off-white solid which was washed with hexane (2×5 mL) to afford the pure product as a white powder (320 mg, 60%). Crystals suitable for X-ray diffraction were grown from a solution of **4** in hexane and benzene at 4 °C.

^{1}H NMR (400.1 MHz, C_6D_6, 300 K): δ (ppm) 7.80 (m, 4H, *o*-Ar-$\underline{H}$), 7.06 (t, 4H, J_{HH} = 7.4 Hz *m*-Ar-$\underline{H}$), 6.85 (t, 2H, J_{HH} = 7.4 Hz, *p*-Ar-$\underline{H}$), 1.47 (s, 12H, N-$C\underline{H}_3$ ×4), 1.33 (br s, 8H, PMDETA $C\underline{H}_2$ ×4), 1.33 (s, 3H, N-$C\underline{H}_3$), 0.61-0.57 (m, 4H, $B(C\underline{H}_2SiMe_3)$ ×2), 0.27 (s, 27H, $SiMe_3$ ×3)

^{13}C{^{1}H} NMR (100.6 MHz, C_6D_6, 300 K): δ (ppm) 133.1 (s, $\underline{C}_{Ph}$), 127.0-126.9 (m. $\underline{C}_{Ph}$), 122.7 (s, $\underline{C}_{Ph}$), 57.0 (s, PMDETA $\underline{C}H_2$×2), 54.6 (s, PMDETA $\underline{C}H_2$×2), 45.4 (s, $N(\underline{C}H_3)_2$ ×2), 44.0 (s, N-$\underline{C}H_3$), 14.1-12.9 (m, $\underline{C}H_2SiMe_3$ ×2), 3.3 (s, $SiMe_3$ ×2). * Signal of C_{Ph}–B not observed.

^{11}B{^{1}H} NMR (128.4 MHz, C_6D_6, 300 K): δ (ppm) −12.9 (s).

Elemental Analysis Calc. for $C_{29}H_{55}BN_3NaSi_2$: C, 65.02; H, 10.35; N, 7.84, found (%): C, 64.83; H, 10.39; N, 8.20.

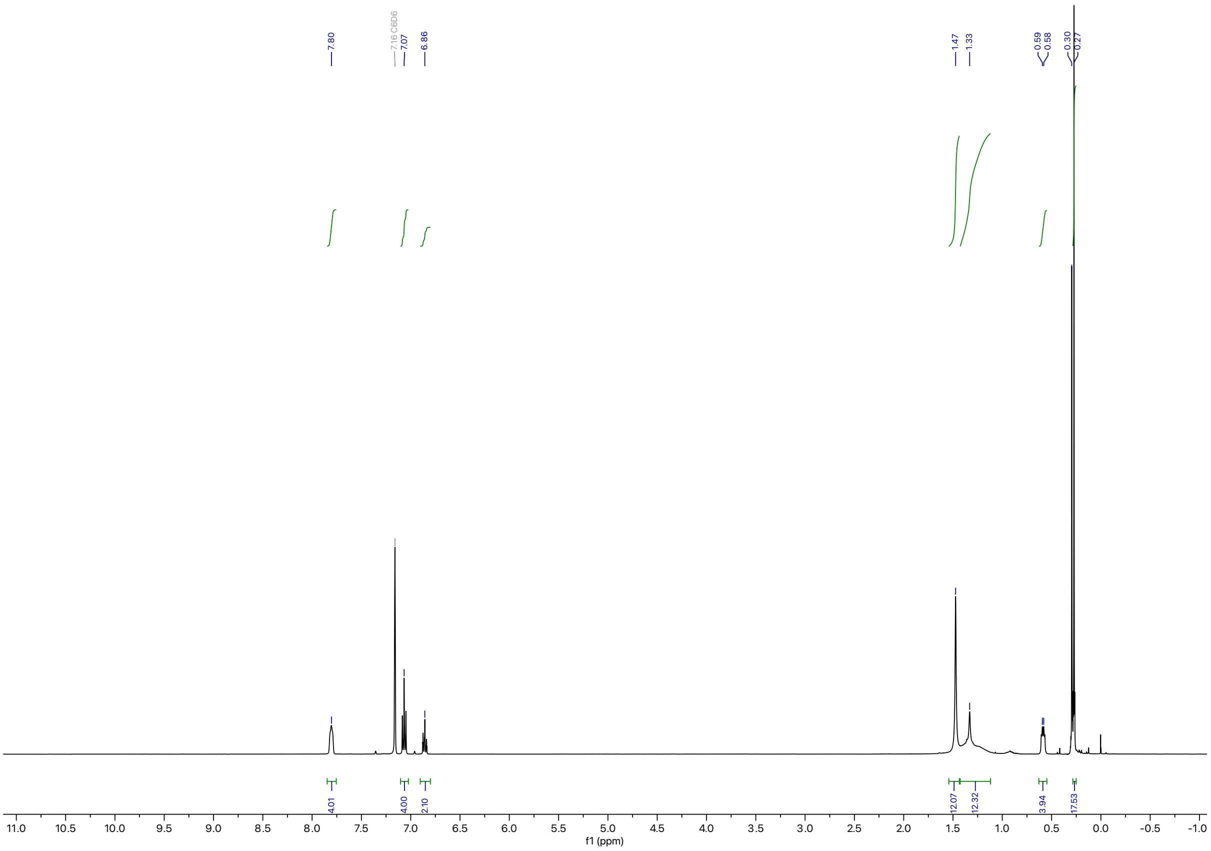

*Figure NMR 10. ^{1}H NMR spectrum of compound **4** in C_6D_6.*

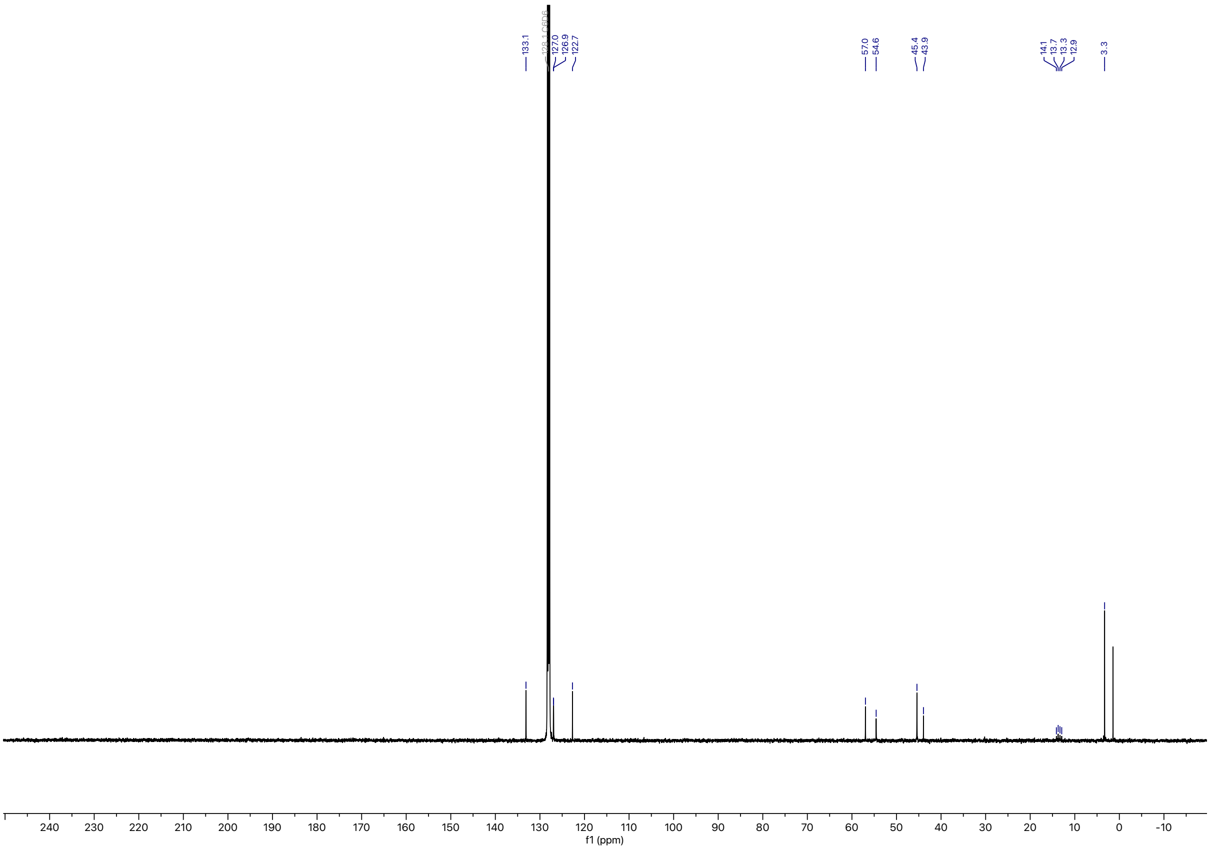

*Figure NMR 11. ^{13}C NMR spectrum of compound **4** in C_6D_6.*

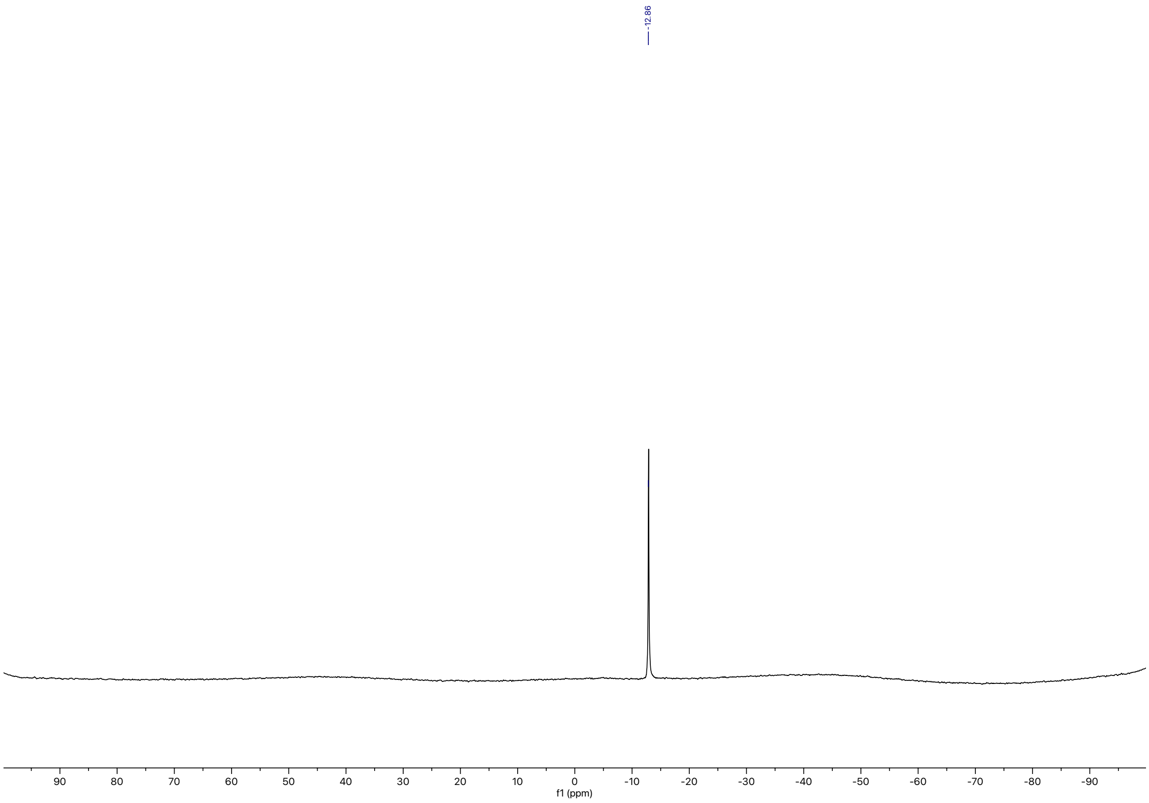

Figure NMR 12. [11]*B NMR spectrum of compound* ***4*** *in* C_6D_6.

- Preparation of **5**

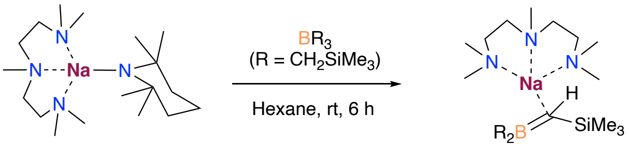

5

Solid NaTMP (163 mg, 1 mmol) was added to a solution of $B(CH_2SiMe_3)_3$ (272 mg, 1 mmol) in hexane (8 mL) affording a white suspension. Then, PMDETA (210 µL,1 mmol) was added giving bright yellow suspension which rapidly transformed to a pale brown solution. The solution was stirred at room temperature for six hours before cooling to −30 °C to afford the product as colourless crystals, which were washed with cold hexane to obtain the pure product (274 mg, 0.59 mmol, 59%).

^{1}H NMR (400.1 MHz, C_6D_6, 300 K): δ (ppm) 1.92 (s, 12H, PMDETA C*H*$_3$ ×4), 1.88 (s, 3H, PMDETA C*H*$_3$ ×1), 1.75 (br s, 8H, PMDETA C*H*$_2$ ×4), 1.71 (s, 1H, C-*H*), 0.80 (s, 2H, BC*H*$_2$SiMe$_3$), 0.42 (s, 9H, SiMe$_3$), 0.42 (s, 9H, SiMe$_3$), 0.39 (s, 2H, BC*H*$_2$SiMe$_3$), 0.31 (s, 9H, SiMe$_3$).

^{13}C{^{1}H} NMR (100.6 MHz, C_6D_6, 300 K): δ (ppm) 62.6 (s, *C*(H)BSi), 57.2 (s, PMDETA *C*H$_2$), 54.0 (s, PMDETA *C*H$_2$), 45.9 (s, PMDETA *C*H$_3$ ×4), 44.3 (s, PMDETA *C*H$_3$ ×1), 19.2 (s, *C*H$_2$) 16.3 (s, *C*H$_2$), 6.3 (s, SiMe$_3$), 2.5 (s, SiMe$_3$), 2.1 (s, SiMe$_3$).

^{11}B{^{1}H} NMR (128.4 MHz, C_6D_6, 300 K): δ (ppm) 49.3 (br).

Elemental Analysis Calc. for $C_{21}H_{55}BN_3NaSi_3$: C, 53.92; H, 11.85; N, 8.98, found (%): C, 53.60; H, 11.59; N, 8.67.

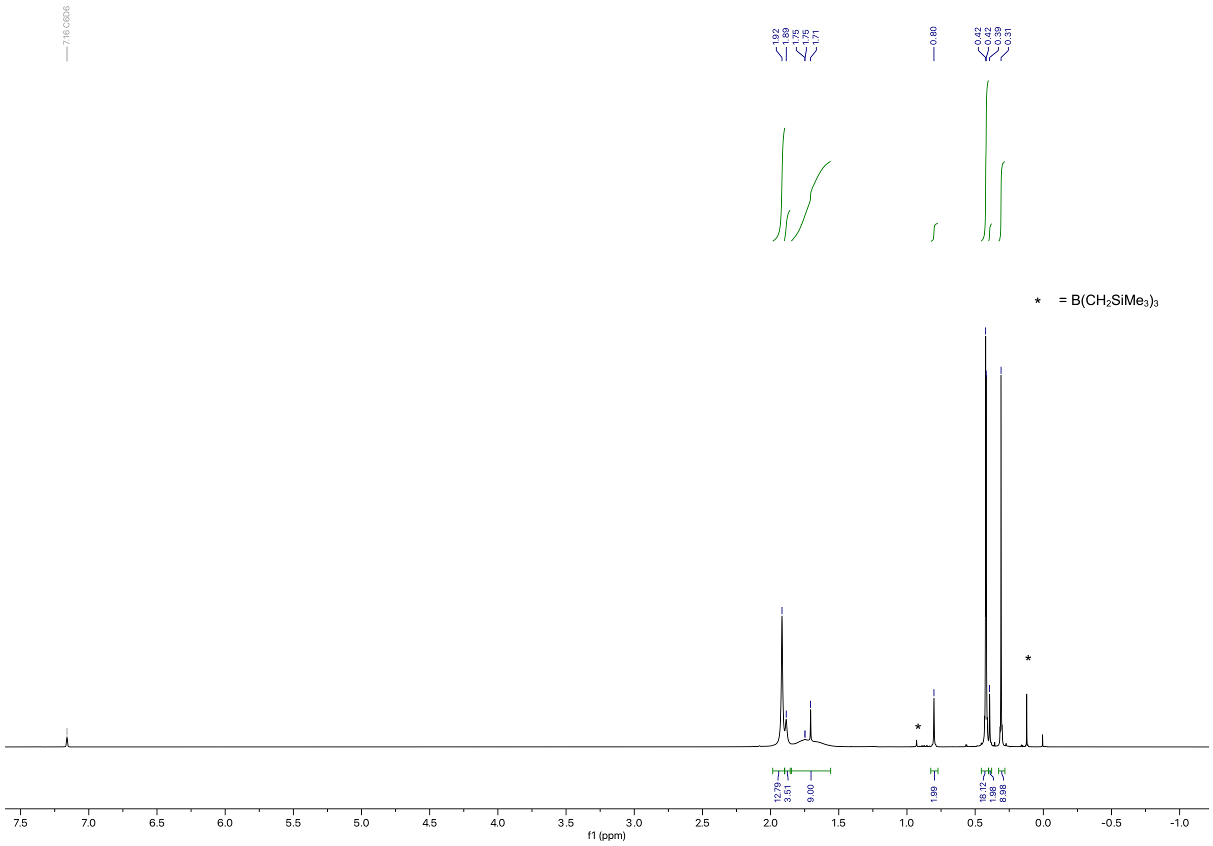

Figure NMR 13. ^{1}H NMR spectrum of compound **5** in C_6D_6.

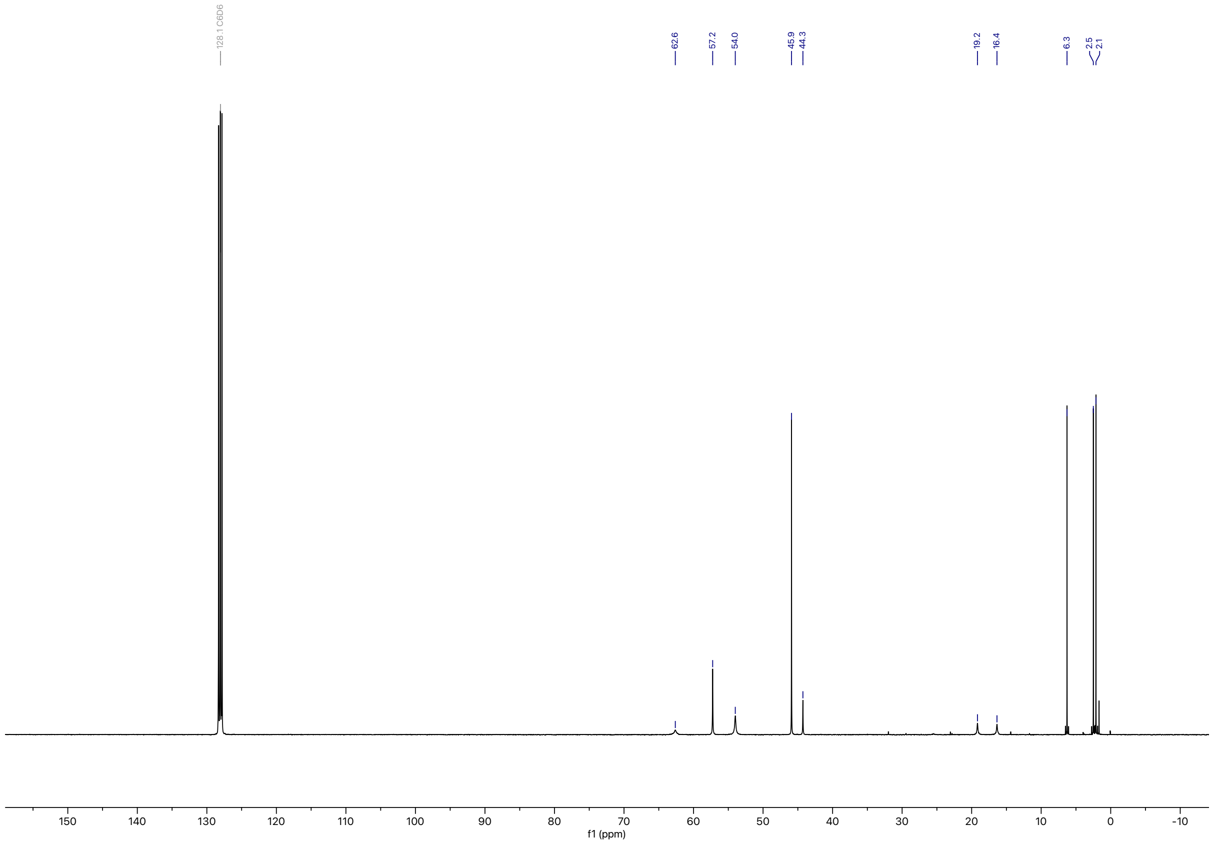

Figure NMR 14. ^{13}C NMR spectrum of compound **5** in C_6D_6.

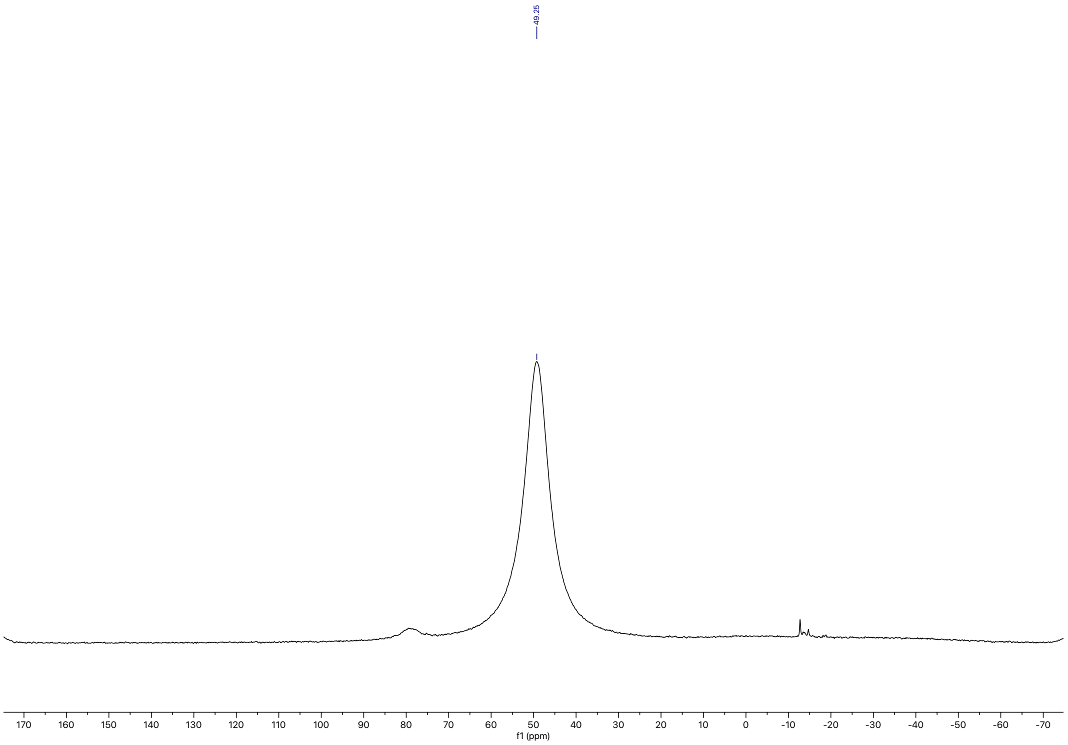

Figure NMR 15. ^{11}*B NMR spectrum of compound* ***5*** *in* C_6D_6*.*

- Preparation of **8**

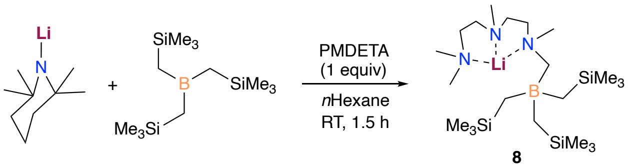

Solid LiTMP (147 mg, 1 mmol) was added to a solution of $B(CH_2SiMe_3)_3$ (272 mg, 1 mmol) in hexane (8 mL) affording a fine suspension. Then, PMDETA (210 µL,1 mmol) was added giving bright yellow suspension which slowly transformed to a yellow solution. The solution was stirred at room temperature for 1.5 hours before concentration by half and cooling to −30 °C to afford the product as colourless crystals which were washed with cold hexane to obtain the product (177 mg, 0.39 mmol, 39%).

^{1}H NMR (400.1 MHz, C_6D_6, 300 K): δ (ppm) 2.34–2.27 (m, 1H, PMDETA C$\underline{H}_2$ ×0.5), 2.05 (s, 3H, PMDETA C$\underline{H}_3$ ×1), 2.03–1.99 (m, 1H, NC$\underline{H}_2$BR$_3$ ×0.5), 1.92–1.80 (m, 3H, PMDETA C$\underline{H}_2$ ×0.5×2, NC$\underline{H}_2$BR$_3$ ×0.5), 1.81 (s, 3H PMDETA C$\underline{H}_3$), 1.78–1.67 (m, 8H, PMDETA C$\underline{H}_2$ ×0.5×2, PMDETA C$\underline{H}_3$ ×2), 1.61–1.49 (m, 3H, PMDETA C$\underline{H}_2$ ×1.5), 0.41 (s, SiMe$_3$ ×3), −0.29–−0.75 (m, 6H, -B(C$\underline{H}_2$SiMe$_3$)$_3$).

^{13}C{^{1}H} NMR (100.6 MHz, C_6D_6, 300 K): δ (ppm) 65.9–64.4 (m, N$\underline{C}$H$_2$BR$_3$), 58.6 (s, PMDETA $\underline{C}$H$_2$), 56.7 (s, PMDETA $\underline{C}$H$_2$), 55.0 (s, PMDETA $\underline{C}$H$_2$), 52.8 (s, PMDETA $\underline{C}$H$_2$), 46.9 (s, PMDETA $\underline{C}$H$_3$), 45.2 (s, PMDETA $\underline{C}$H$_3$), 44.9 (s, PMDETA $\underline{C}$H$_3$), 44.3 (s, PMDETA $\underline{C}$H$_3$), 16.2−15.1 (m, B($\underline{C}$H$_2$SiMe$_3$)$_3$), 4.0 (s, SiMe$_3$ ×3).

^{11}B{^{1}H} NMR (128.4 MHz, C_6D_6, 300 K): δ (ppm) –15.1 (s).

Elemental Analysis Calc. for $C_{21}H_{55}BN_3LiSi_3$: C, 55.84; H, 12.27; N, 9.30. Found (%): C, 56.0; H, 12.54; N, 9.56.

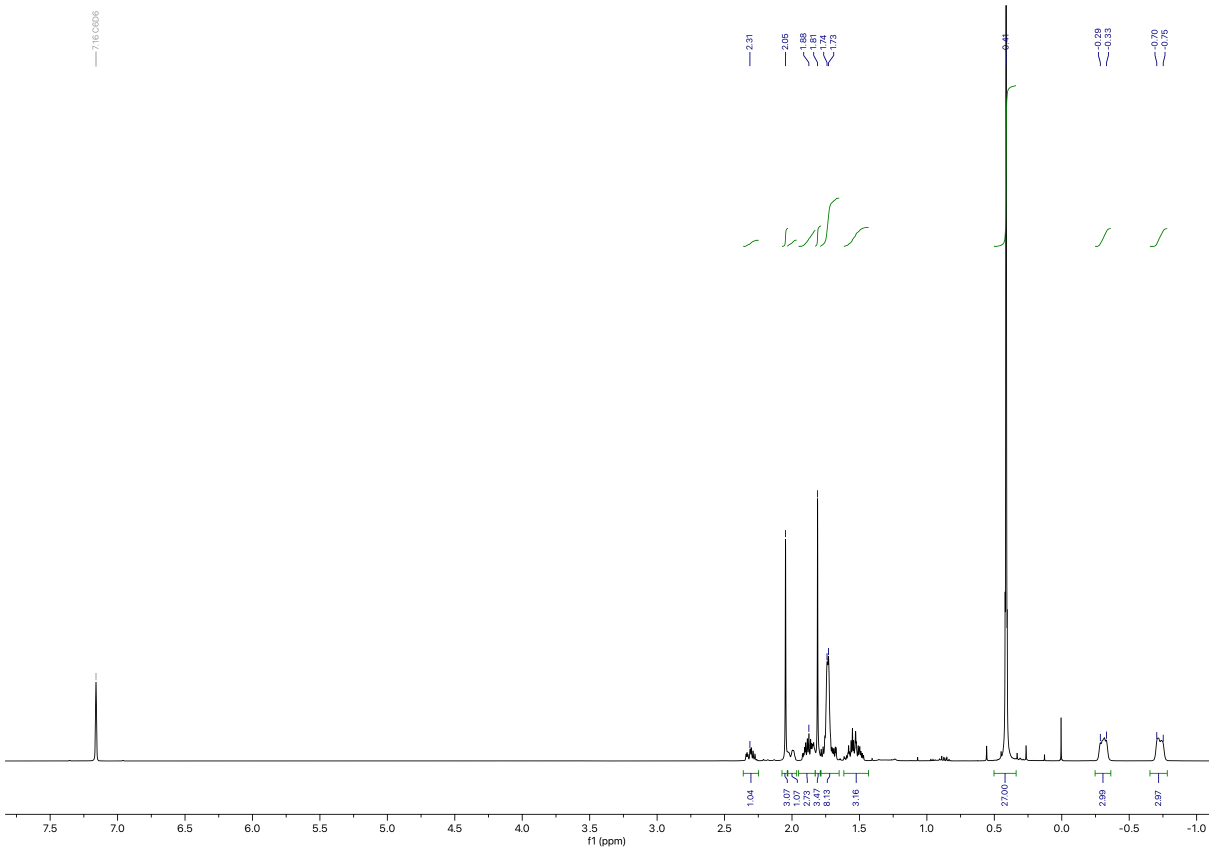

Figure NMR 16. [1]*H NMR spectrum of compound* ***8*** *in* C_6D_6.

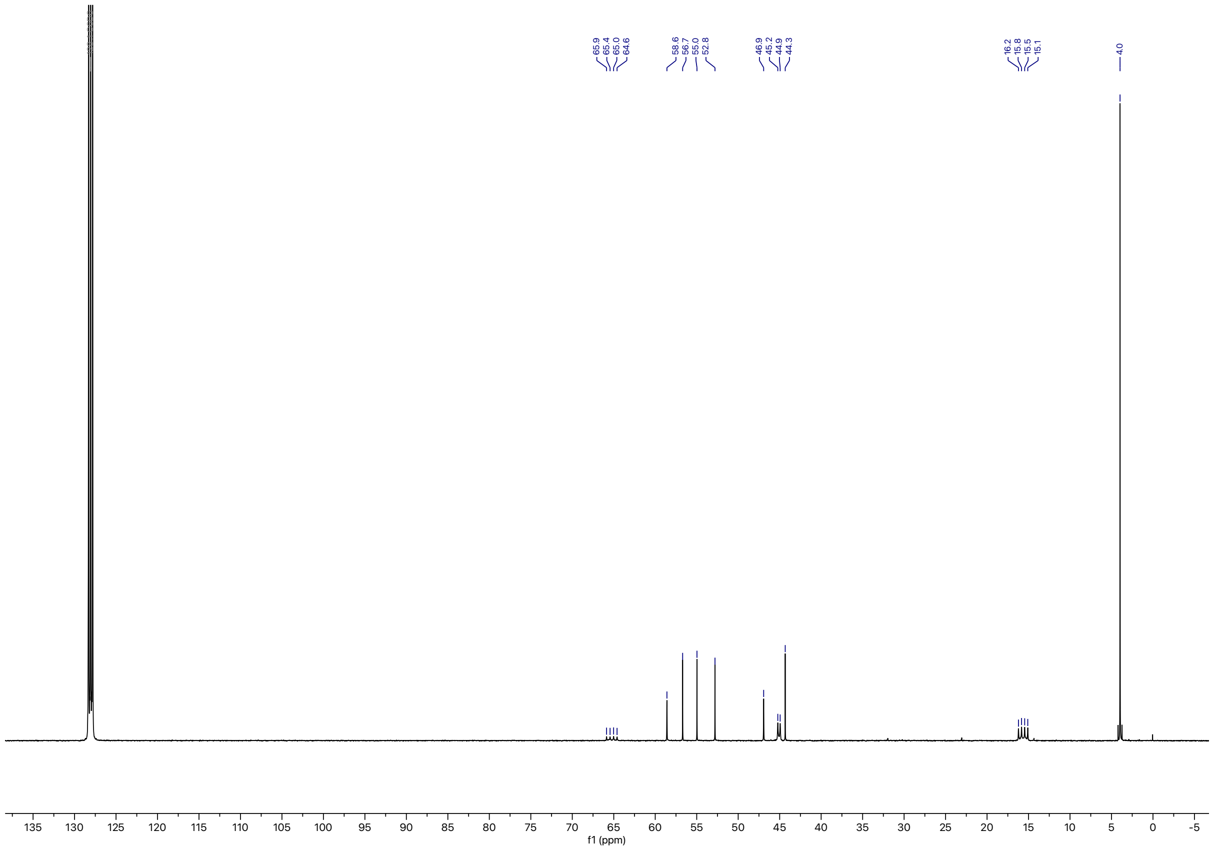

Figure NMR 17. [13]*C NMR spectrum of compound* ***8*** *in* C_6D_6.

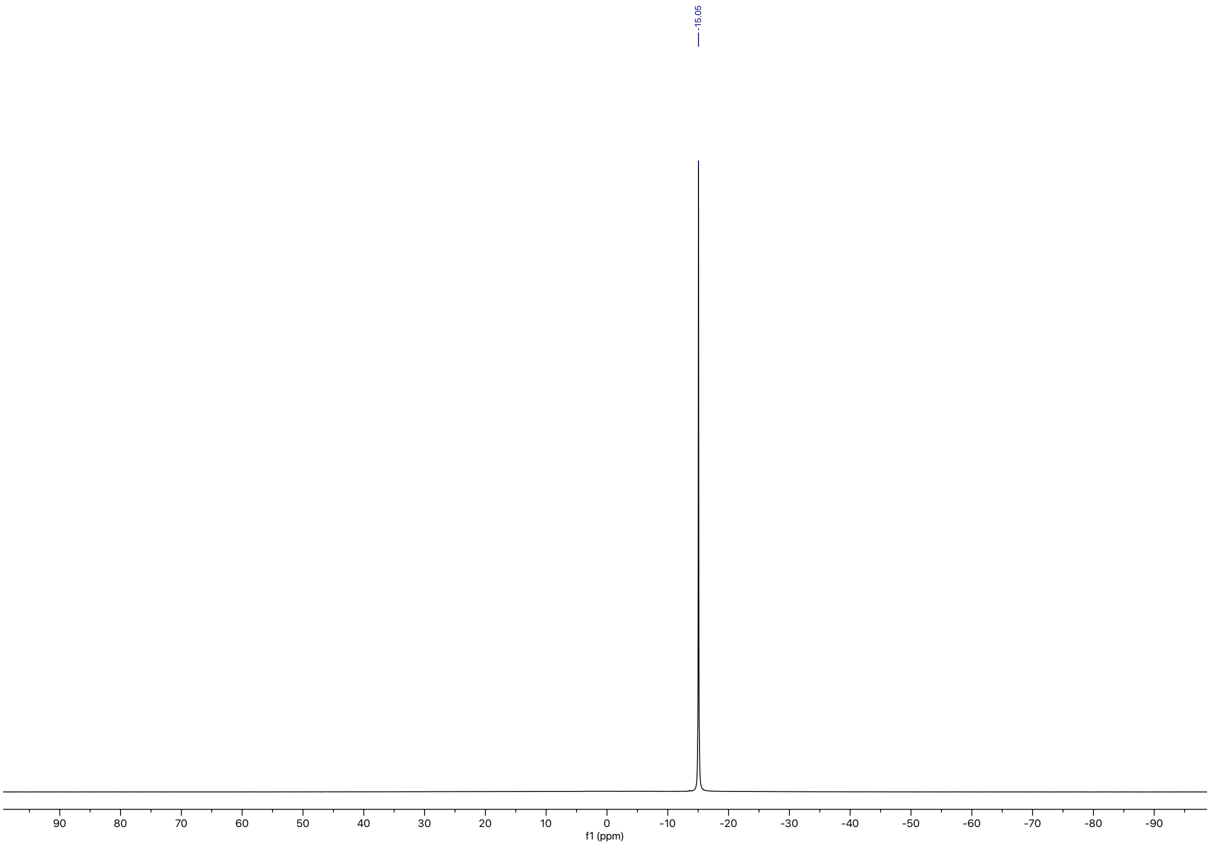

*Figure NMR 18. ^{11}B NMR spectrum of compound **8** in C_6D_6.*

- Reactivity with compound **5**

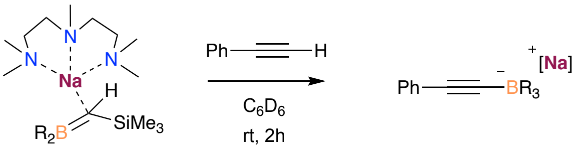

5 **6**

In an argon-filled glovebox, compound **5** (0.025 mmol, 11.3 mg) is dissolved in C_6D_6 in an J-Young NMR tube. To this solution, phenylacetylene (0.025 mmol, 2.8 µL) is added, and the reaction kept at room temperature for 2 hours. A white solid precipitates forms, which can be partially dissolved by adding a few drops of THF. ^{1}H, ^{13}C and ^{11}B NMR spectra were recorded, showing the formation of a new borate, assigned as compound **6**, with the formation of some free BR_3 in solution and some unreacted PhCCH. Due to the poor solubility of **6**, the ^{13}C NMR spectrum was not sensitive enough to detect the signal of some quaternary carbons.

^{1}H NMR (300 MHz, C_6D_6, 300K) δ (ppm) = 7.46 – 7.40 (m, 2H, C_{Ph}-H), 7.00 (m, 1H, C_{Ph}-H), 6.94 – 6.89 (m, 2H, C_{Ph}-H), 1.91 (s, 23H, PMDETA), 0.52 (s, 27H, $SiCH_3$), -0.13 (s, 6H, BCH_2TMS).

^{11}B NMR (96 MHz, C_6D_6, 300K) δ (ppm) = -19.4.

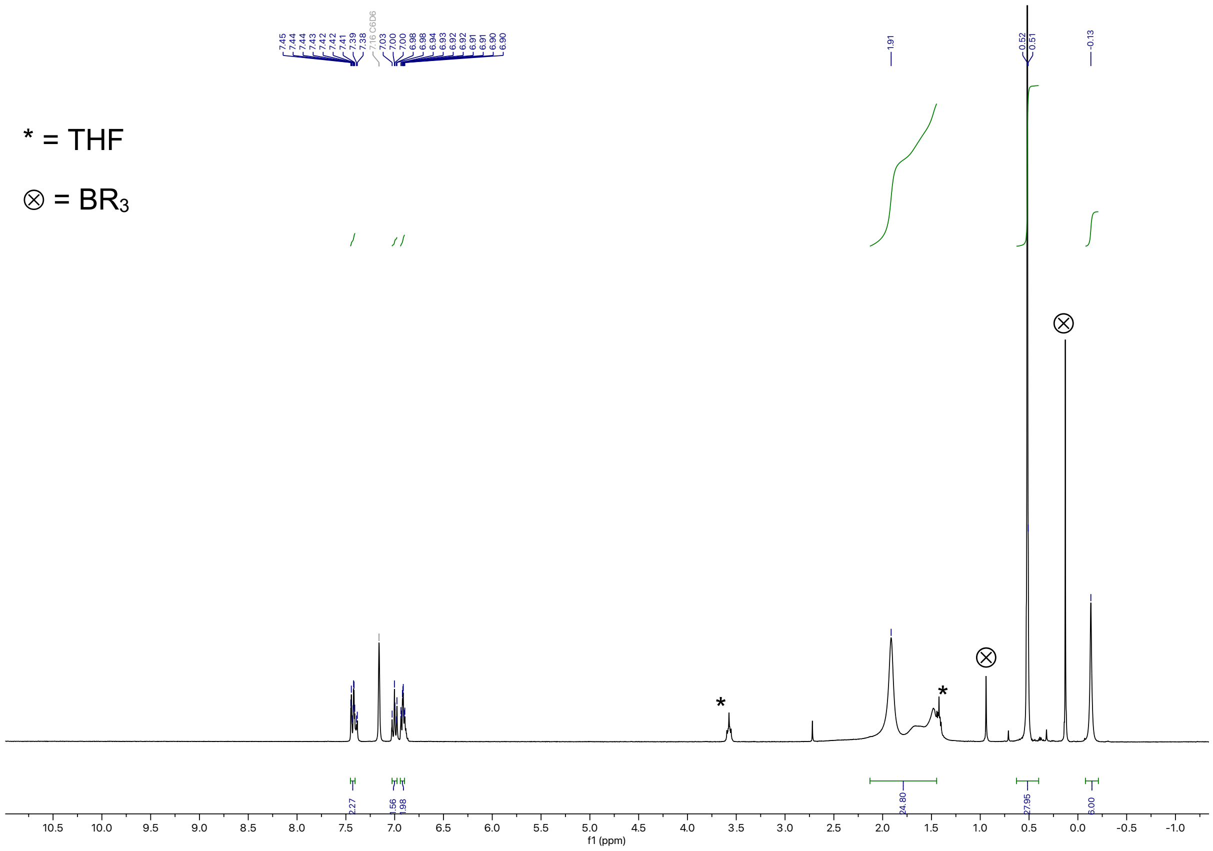

*Figure NMR 19. 1H NMR spectrum of the reaction of **5** with phenylacetylene to produce **6**.*

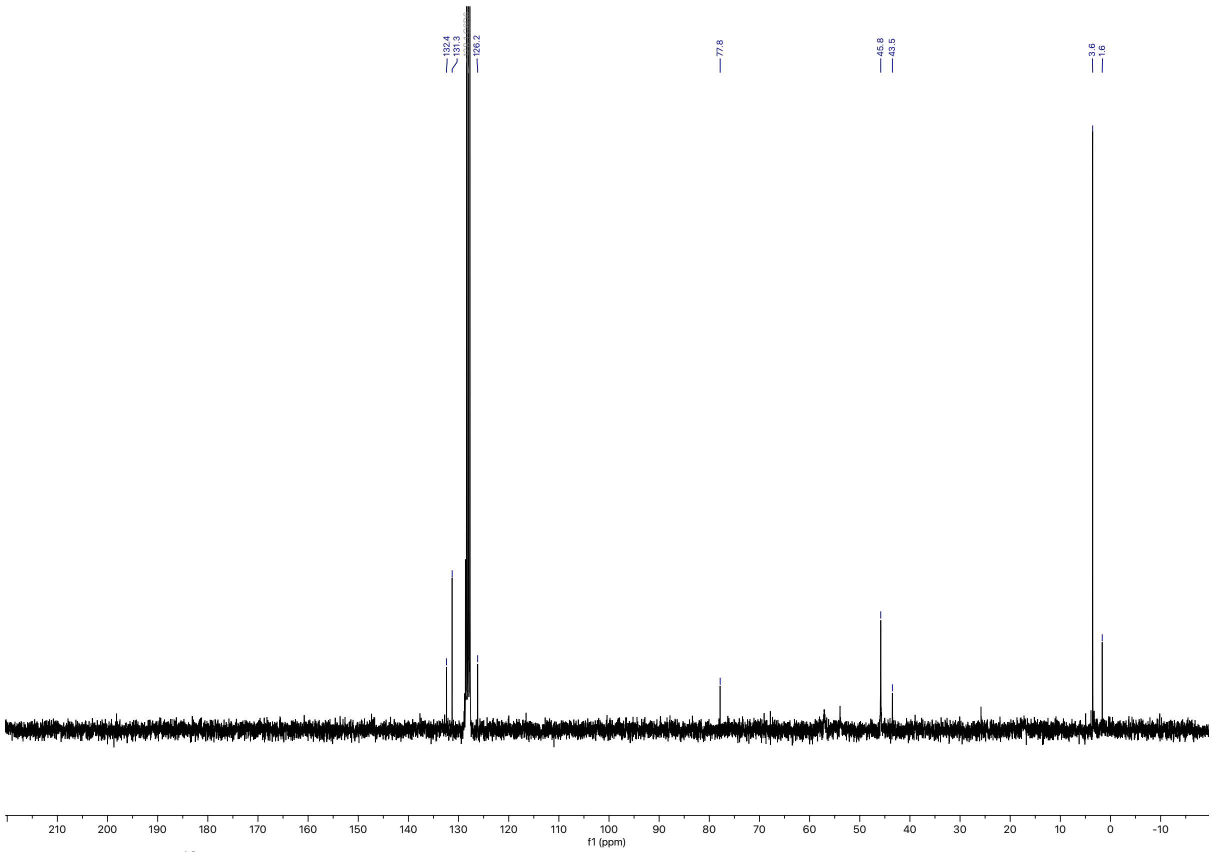

Figure NMR 20. [13]*C NMR spectrum of the reaction of **5** with phenylacetylene to produce **6**.*

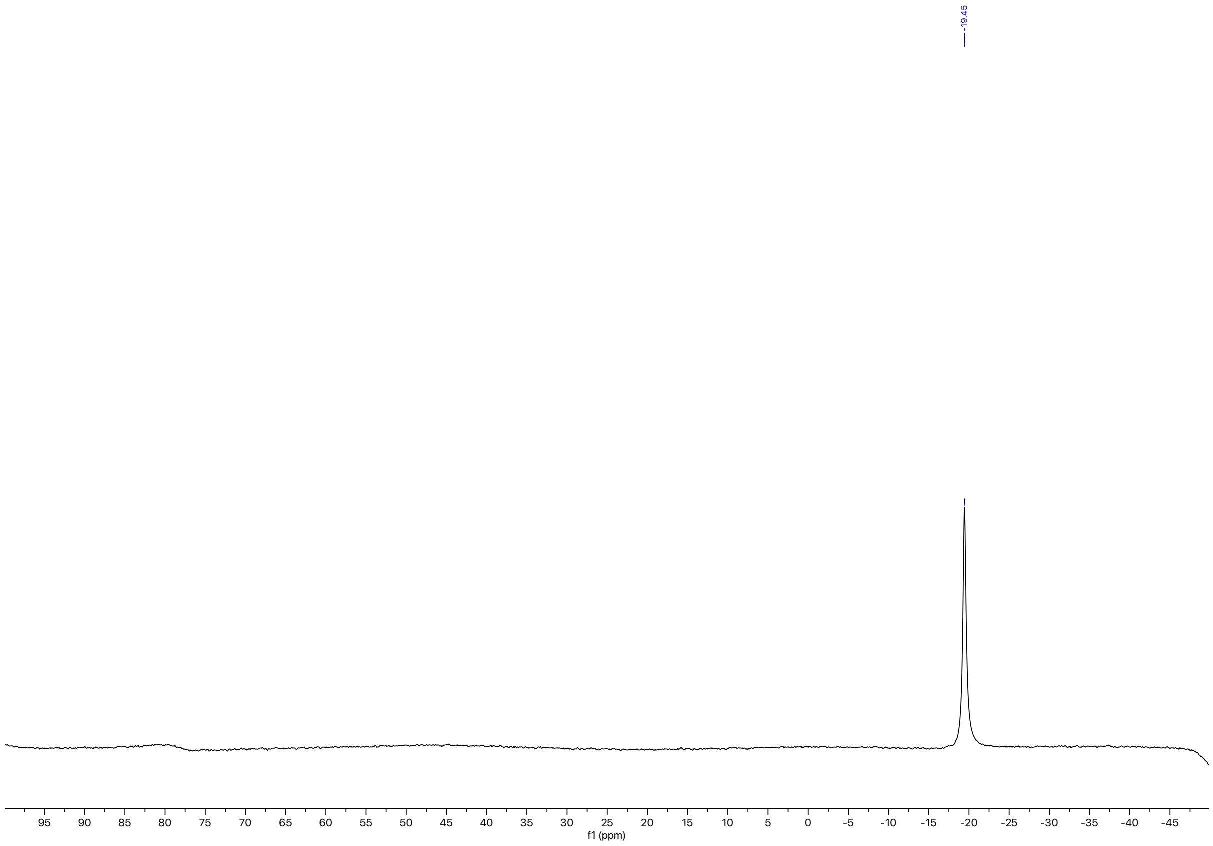

*Figure NMR 21. ^{11}B NMR spectrum of the reaction of **5** with phenylacetylene to produce **6**.*

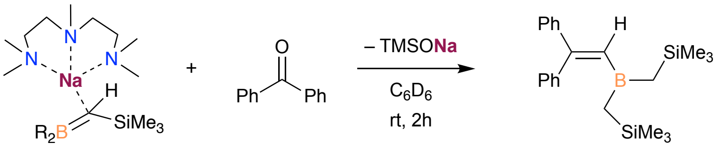

5 **7**

In an argon-filled glovebox, compound **5** (0.05 mmol, 23.4 mg) is dissolved in C_6D_6 in an J-Young NMR tube. To this solution, benzophenone (0.06 mmol, 10.9 mg) is added, and the reaction kept at room temperature for 2 hours. ^{1}H, ^{13}C and ^{11}B NMR spectra were recorded. Full conversion of compound is observed by ^{1}H and ^{11}B NMR. ^{1}H-^{1}H COSY, ^{1}H-^{13}C HSQC and HMBC and ^{13}C-APT were also recorded to confirm the molecular structure of compound **7** in solution.

^{1}H NMR (300 MHz, C_6D_6, 300 K) δ (ppm) 7.45 – 7.39 (m, 2H, C_{Ph}-*H*), 7.26 – 7.02 (m, 8H, C_{Ph}-*H*), 6.72 (s, 1H, C=C-*H*), 2.48 (d, *J* = 6.8 Hz, 4H, CH_2-PMDETA), 2.44 – 2.32 (m, 4H, CH_2-PMDETA), 2.19 (s, 3H, CH_3-PMDETA), 2.12 (s, 12H, CH_3-PMDETA), 0.95 (s, 4H. CH_2-SiMe$_3$), 0.10 (s, 18H, SiCH_3).

^{13}C NMR (75 MHz, C_6D_6, , 300 K) δ (ppm) 156.3 (Ph$_2$<u>C</u>=(CH)B), 144.6(<u>C</u>$_{q\text{-}Ph}$), 142.9(<u>C</u>$_{q\text{-}Ph}$), 138.2 (*br* s, Ph$_2$C=(<u>C</u>H)B), 130.8 (<u>C</u>$_{Ar}$–H), 128.7(<u>C</u>$_{Ar}$–H), 128.5(<u>C</u>$_{Ar}$–H), 128.2(<u>C</u>$_{Ar}$–H), 128.0(<u>C</u>$_{Ar}$–H), 58.4 (<u>C</u>H$_2$-PMDETA), 56.9(<u>C</u>H$_2$-PMDETA), 46.1(<u>C</u>H$_3$-PMDETA), 43.2(<u>C</u>H$_3$-PMDETA), 26.4 (B-<u>C</u>H$_2$SiMe$_3$), 1.3 (Si<u>C</u>H$_3$). *1 Aromatic carbon overlap with the signal of the solvent.

^{11}B{^{1}H} NMR (128.4 MHz, C_6D_6, 300 K): δ (ppm) 73.3 (*br* s).

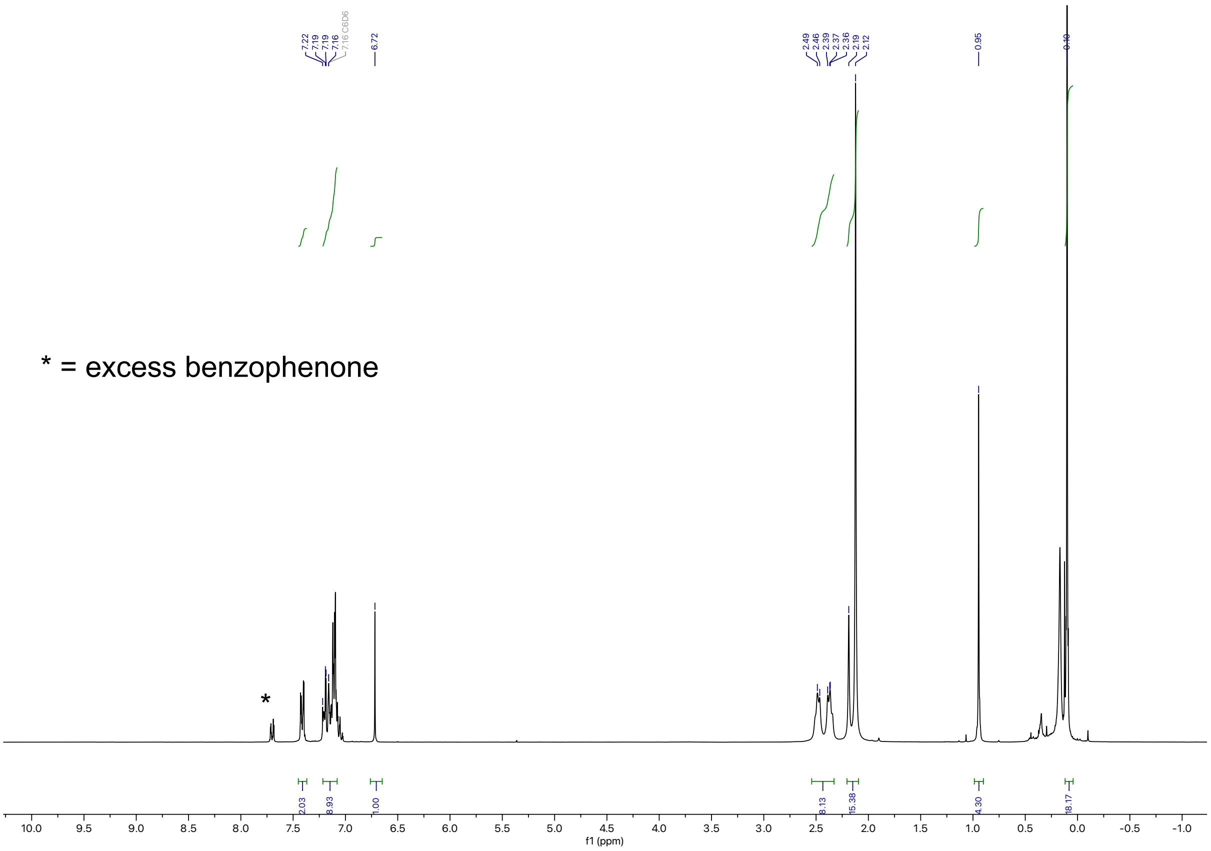

*Figure NMR 22. ^{1}H NMR of the reaction of compound **5** with benzophenone to form **7**.*

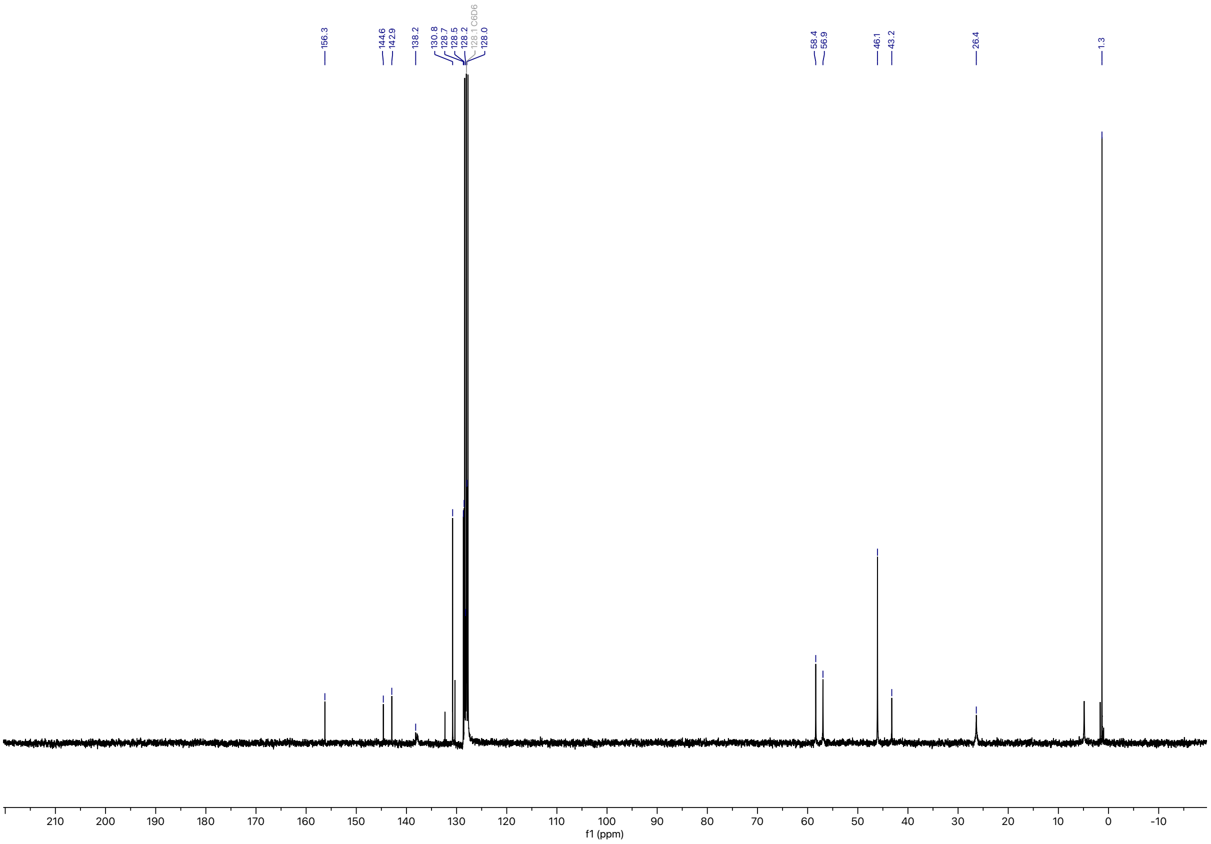

*Figure NMR 23. ^{13}C NMR of the reaction of compound **5** with benzophenone to form **7**.*

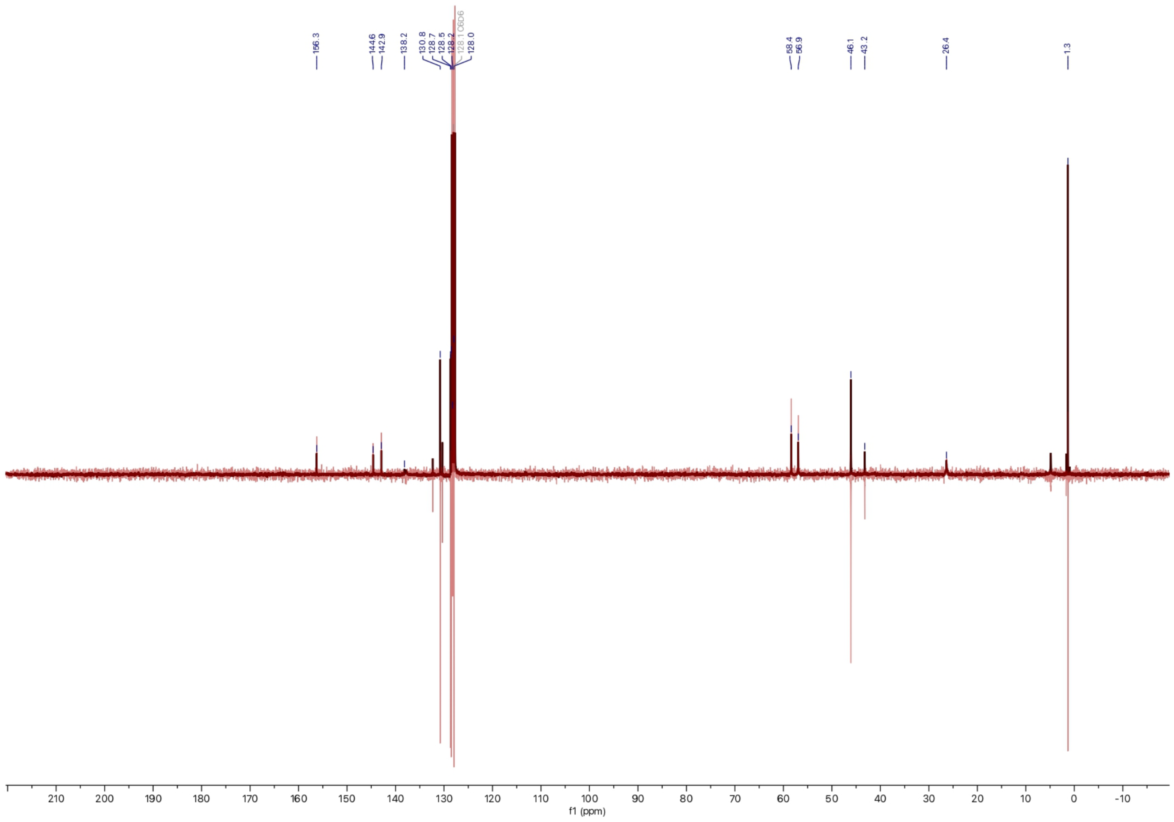

*Figure NMR 24. ^{13}C NMR(regular and APT) of the reaction of compound **5** with benzophenone to form **7**.*

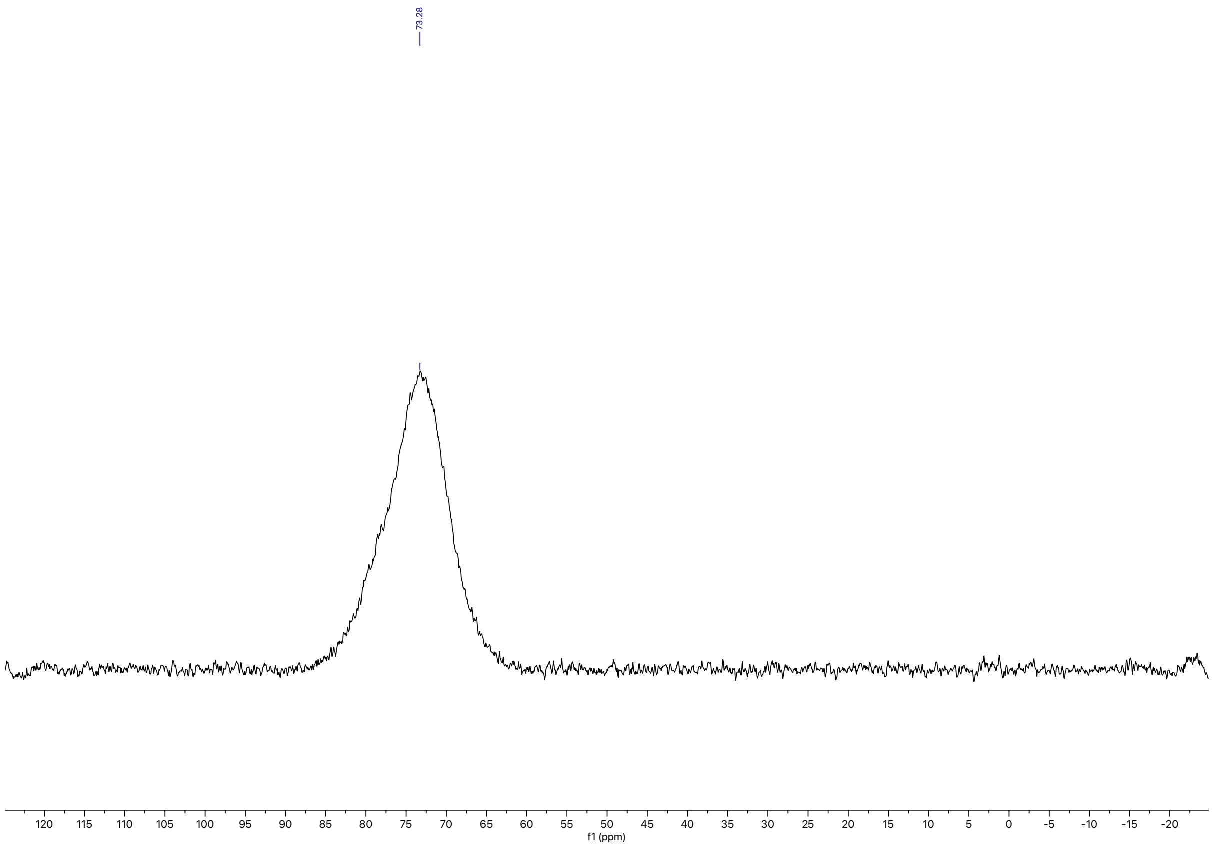

*Figure NMR 25. ^{11}B NMR of the reaction of compound **5** with benzophenone to form **7**.*

- Control experiments with compound **1**

To rule out the deprotonation of compound **1** by an in situ formed (PMDETA)$NaCH_2SiMe_3$, we reacted compound **1** (0.025 mmol, 14.4 mg) with $NaCH_2SiMe_3$ (0.025 mmol. 2.7 mg) and PMDETA (0.025 mmol, 5.3 μL) in C_6D_6. By ^1H NMR and ^{11}B NMR spectroscopy we observed that both components did not react at room temperature, ruling out this mechanistic pathway in the formation of compound **2** from **1**.

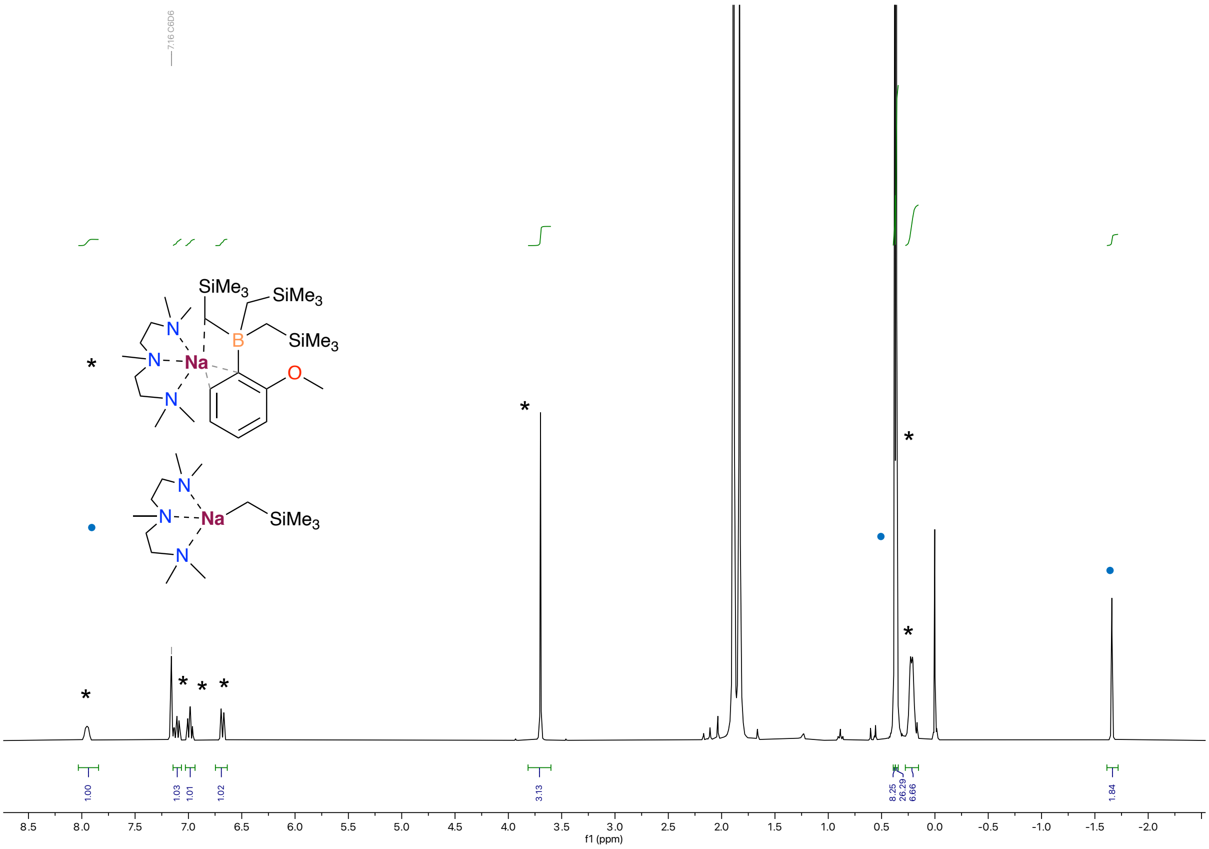

Figure NMR 24.^1H *NMR of the reaction of compound* ***1*** *with (PMDETA)*$NaCH_2SiMe_3$*.*

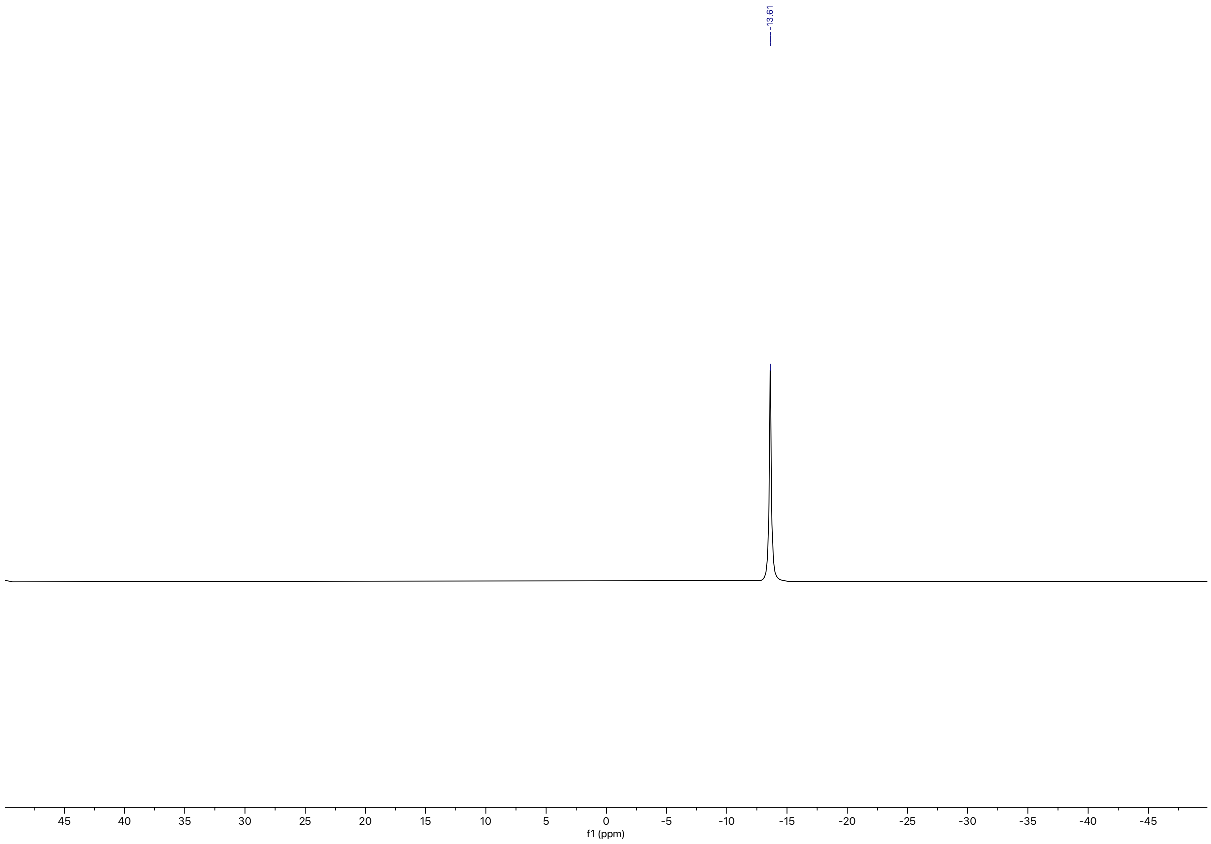

*Figure NMR 25.^{11}B NMR of the reaction of compound **1** with (PMDETA)NaCH$_2$SiMe$_3$.*

X-Ray Crystallographic Details

Compound **1**: Crystallographic data for [(PMDETA)NaB(C_6H_4-OMe)(CH_2SiMe_3)$_3$] (CCDC 2245605). A crystal of $C_{28}H_{63}BN_3NaOSi_3$ immersed in parabar oil was mounted at ambient conditions and transferred into the stream of nitrogen (173 K). All measurements were made on a *RIGAKU Synergy S* area-detector diffractometer[2] using mirror optics monochromated Cu *K*α radiation (λ = 1.54184 Å). The unit cell constants and an orientation matrix for data collection were obtained from a least-squares refinement of the setting angles of reflections in the range 5.102° < 2θ < 154.984°. A total of 2874 frames were collected using ω scans, with 0.3 second exposure time (1 s for high-angle reflections), a rotation angle of 0.5° per frame, a crystal-detector distance of 34.0 mm, at T = 173(2) K.

Compound **2**: Crystallographic data for [(PMDETA)NaB(C_6H_4-OMe)$_2$(CH_2SiMe_3)$_2$] (CCDC 2245606). A crystal of $C_{31}H_{59}BN_3NaO_2Si_2$ (about 70%) and $C_{30}H_{57}BN_3NaO_1Si_2$ (about 30%) immersed in parabar oil was mounted at ambient conditions and transferred into the stream of nitrogen (100 K). All measurements were made on a *RIGAKU Synergy S* area-detector diffractometer[2] using mirror optics monochromated Cu *K*α radiation (λ = 1.54184 Å). The unit cell constants and an orientation matrix for data collection were obtained from a least-squares refinement of the setting angles of reflections in the range 2.692° < θ < 79.649°. A total of 2606 frames were collected using ω scans, with 0.15 second exposure time (0.8 s for high-angle reflections), a rotation angle of 0.5° per frame, a crystal-detector distance of 34.0 mm, at T = 100(2) K.

The structure contains dynamic disorder and substitutional disorder where a PhOMe group is exchanged by Ph.

Compound **3**: Crystallographic data for [(PMDETA)NaB(C_6H_5)(CH_2SiMe_3)$_3$] (CCDC 2245607). A crystal of $C_{27}H_{61}BN_3NaSi_3$ immersed in parabar oil was mounted at ambient conditions and transferred into the stream of nitrogen (100 K). All measurements were made on a *RIGAKU Synergy S* area-detector diffractometer[2] using mirror optics monochromated Cu *K*α radiation (λ = 1.54184 Å). The unit cell constants and an orientation matrix for data collection were obtained from a least-squares refinement of the setting angles of reflections in the range 7.444° < 2θ < 159.222°. A total of 164 frames were collected using ω scans, with 0.2 second exposure time, a rotation angle of 0.5° per frame, a crystal-detector distance of 34.0 mm, at T = 100(2) K.

Compound **4**: Crystallographic data for [(PMDETA)NaB(C_6H_5)$_2$(CH_2SiMe_3)$_2$] (CCDC 2245608). A crystal of $C_{29}H_{55}BN_3NaSi_2$ immersed in parabar oil was mounted at ambient conditions and transferred into the stream of nitrogen (100 K). All measurements were made on a *RIGAKU Synergy S* area-detector diffractometer[2] using mirror optics monochromated Cu *K*α radiation (λ = 1.54184 Å). The unit cell constants and an orientation matrix for data collection were obtained from a least-squares refinement of the setting angles of reflections in the range 7.224° < 2θ < 154°. A total of 1522 frames were collected using ω scans, with 0.5 second exposure time (1 s for high-angle reflections), a rotation angle of 0.5° per frame, a crystal-detector distance of 34.0 mm, at T = 100(2) K.

Compound **5**: Crystallographic data for [(PMDETA)NaCH($SiMe_3$)B(CH_2SiMe_3)$_2$] (CCDC 2245609). A crystal of $C_{21}H_{55}BN_3NaSi_3$ immersed in parabar oil was mounted at ambient conditions and transferred into the stream of nitrogen (173 K). All measurements were made on a *RIGAKU Synergy S* area-detector diffractometer[2] using mirror optics monochromated Cu *K*α radiation (λ = 1.54184 Å). The unit cell constants and an orientation matrix for data collection were obtained from a least-squares refinement of the setting angles of reflections in the range 7.784° < 2θ < 153.774°. A total of 3540 frames were collected using ω scans, with 0.1 second exposure time (0.25 s for high-angle reflections), a rotation angle of 0.5° per frame, a crystal-detector distance of 34.0 mm, at T = 173(2) K.

Solvent accessible areas were found however these voids do not contain any electron density to be modeled.

Compound **8**: Crystallographic data for [(PMDETA$^-$)LiB(CH_2SiMe_3)$_3$] (CCDC 2245610). A crystal of $C_{21}H_{55}BLiN_3Si_3$ was mounted in mineral oil under inert conditions and inserted into the protecting nitrogen gas cooling stream. The measurement was conducted on a *RIGAKU Synergy S* area-detector diffractometer[2] using mirror-optics monochromated Cu *K*α radiation (λ = 1.54184 Å). The measurement was carried out in a resolution range 9.73° < θ < 160.48°. A total of 6656 symmetry-independent reflections were collected using ω scans, a rotation angle of 0.5° per frame, a crystal-detector distance of 34.0 mm, at T = 173.00(10) K.

Some of the hydrogen atoms in the vicinity of the Li atom could not be fixed geometrically but were found by difference Fourier synthesis and then refined freely. Solvent accessible areas were found however these voids do not contain any electron density to be modeled.

For all compounds: Data reduction was performed using the *CrysAlisPro*[2] program. The intensities were corrected for Lorentz and polarization effects, and an absorption correction based on the multi-scan method using SCALE3 ABSPACK in *CrysAlisPro*[1] was applied. Data collection and refinement parameters are given in *Table S1 and S2.*

The structure was solved by intrinsic phasing using *SHELXT*[3], which revealed the positions of all non-hydrogen atoms of the title compound. All non-hydrogen atoms were refined anisotropically. H-atoms were assigned in geometrically calculated positions and refined using a riding model where each H-atom was assigned a fixed isotropic displacement parameter with a value equal to 1.2Ueq of its parent atom (1.5Ueq for methyl groups).

Refinement of the structure was carried out on F^2 using full-matrix least-squares procedures, which minimized the function $\Sigma w(F_o^2 - F_c^2)^2$. The weighting scheme was based on counting statistics and included a factor to downweight the intense reflections. All calculations were performed using the *SHELXL-2014/7*[4] program in OLEX2.[5]

Disorder model was used for parts of the structure where the occupancies of each disorder component was refined through the use of a free variable. The sum of equivalent components was constrained to 1, i.e. 100%.

__Table S1.__ Selected crystallographic parameters of compounds __1__, __2__ and __3__.

Compound	1	2	3
**CCDC Number**	2245605	2245606	2245607
**Empirical formula**	$C_{28}H_{63}BN_3NaOSi_3$	$C_{31}H_{59}BN_3NaO_2Si_2$	$C_{27}H_{61}BN_3NaSi_3$
**Mol. Mass**	575.88	595.79	545.85
**Temperature/K**	173.01(10)	99.99(10)	100.35(10)
**Crystal system**	monoclinic	orthorhombic	monoclinic
**Space group**	$P2_1/n$	Pbca	$P2_1/n$
**a/Å**	10.11080(10)	11.64110(10)	12.5688(15)
**b/Å**	34.6407(2)	18.66620(10)	17.320(2)
**c/Å**	11.54530(10)	33.6871(2)	16.3239(10)
**α/°**	90	90	90
**β/°**	112.0050(10)	90	91.481(7)
**γ/°**	90	90	90
**V/Å^3**	3749.11(6)	7320.04(9)	3552.4(7)
**Z**	4	8	4
**□/Å**	1.54184	1.54184	1.54184
**ρ_{calc}g/cm^3**	1.020	1.081	1.021
**μ/mm^{-1}**	1.437	1.210	1.473
**F(000)**	1272.0	2608.0	1208.0
**Crystal size/mm^3**	0.15 × 0.12 × 0.05	0.542 × 0.374 × 0.317	0.184 × 0.137 × 0.108
**Reflection collected**	36567	57708	33958
**Unique reflections**	7760	7500	7509
***R*int**	0.0314	0.0386	0.0838
**Goof**	1.066	1.136	1.058
**Final R indexes [I>=2σ (I)]**	R_1 = 0.0360, wR_2 = 0.0959	R_1 = 0.0459, wR_2 = 0.1074	R_1 = 0.0744, wR_2 = 0.1951
**Final R indexes [all data]**	R_1 = 0.0432, wR_2 = 0.1028	R_1 = 0.0469, wR_2 = 0.1080	R_1 = 0.0894, wR_2 = 0.2148
**Largest diff. peak/hole / e Å^{-3}**	0.25/-0.22	0.29/-0.26	0.57/-0.45

***Table S2** Selected crystallographic parameters of compounds **4**, **5** and **8**.*

Compound	4	5	8
**CCDC Number**	2245608	2245609	2245610
**Empirical formula**	$C_{29}H_{55}BN_3NaSi_2$	$C_{21}H_{55}BN_3NaSi_3$	$C_{21}H_{55}BLiN_3Si_3$
**Mol. Mass**	535.74	467.75	451.70
**Temperature/K**	100.3(5)	173.00(10)	173.00(10)
**Crystal system**	monoclinic	monoclinic	monoclinic
**Space group**	$P2_1/n$	$P2_1/n$	Cc
**a/Å**	11.08170(10)	12.50294(10)	10.09239(18)
**b/Å**	18.6842(2)	16.73895(15)	17.0772(3)
**c/Å**	16.2208(2)	15.65536(13)	18.2578(3)
**α/°**	90	90	90
**β/°**	93.3580(10)	98.9971(8)	95.5490(17)
**γ/°**	90	90	90
**V/Å^3**	3352.79(6)	3236.14(5)	3131.98(10)
**Z**	4	4	4
**□/Å**	1.54184	1.54184	1.54184
**ρ_{calc}g/cm^3**	1.061	0.960	0.958
**μ/mm^{-1}**	1.227	1.549	1.456
**F(000)**	1176.0	1040.0	1008.0
**Crystal size/mm^3**	0.16 × 0.095 × 0.055	0.21 × 0.09 × 0.018	0.254 × 0.147 × 0.125
**Reflection collected**	32490	37617	31296
**Unique reflections**	6931	6649	6654
***R*int**	0.0682	0.0447	0.0635
**Goof**	1.071	1.069	1.126
**Final R indexes [I>=2σ (I)]**	R_1 = 0.0549, wR_2 = 0.1474	R_1 = 0.0511, wR_2 = 0.1415	R_1 = 0.0479, wR_2 = 0.1382
**Final R indexes [all data]**	R_1 = 0.0643, wR_2 = 0.1574	R_1 = 0.0577, wR_2 = 0.1492	R_1 = 0.0501, wR_2 = 0.1436
**Largest diff. peak/hole / e Å^{-3}**	0.59/-0.55	0.61/-0.44	0.30/-0.16
**Flack parameter**	----	----	0.03(3)

Computational Methods

The mechanisms for the C–H metalation of C_6F_5H were elucidated with density functional theory (DFT) calculations by employing the dispersion-corrected hybrid exchange-correlation functional ωb97xd,[6] as implemented in the Gaussian09 software.[7] The Na, Li, Si, B, C, H atoms were modelled with double-zeta 6-31g(d, p) basis set. The same basis set plus diffuse *d*-functions, 6-31+g(d), was employed to describe the more electronegative N and O atoms. Geometry optimisations were carried out in vacuum without imposing any symmetry constraints as closed shell systems.

The nature of the stationary points was confirmed by vibrational frequency analysis, wherein energy minima were confirmed to display only real frequencies, and transition states exhibited a single imaginary frequency corresponding to the desired reaction coordinates. The optimised transition state structures were also relaxed in both directions along the reaction coordinate to confirm that they connect the expected energy minima. The effect of the solvent employed in experiments (n-hexane and anisole, ε = 1.8819 and 4.2247, respectively) was included via single-point calculations at the optimised structures in vacuum using the SMD continuum solvation model.[8] Standard state corrections were applied by adding (subtracting) 1.90 kcal/mol to the computed reaction Gibbs energies for every additional molecule with respect to the products (reactants).[9]

Gibbs energies reported in this work were calculated at the experimental temperature and pressures of 298.15 or 353.15 K and 1 atm, and are provided in kcal/mol. The electron density and Laplacian values at the bond critical points were calculated by means of quantum theory of atoms in molecules (QTAIM)[10] using the Multiwfn software.[11] Natural bonding orbital (NBO) analysis was performed using the converged wavefunction and structure via NBO 7.0.[12]

Gibbs energy profile for the Formation of Complex 1

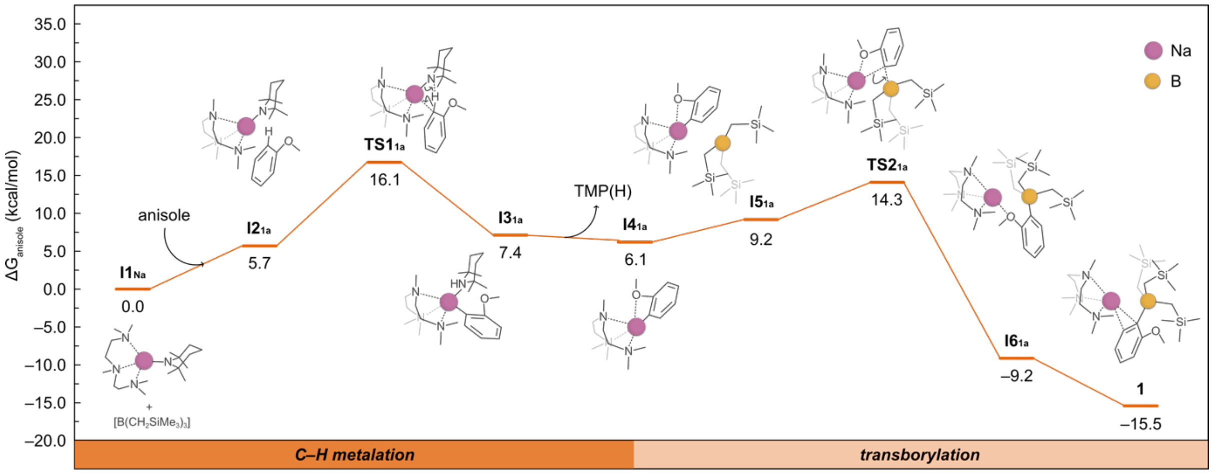

*Figure S1. Gibbs energy profile for the deprotonative borylation of anisole with [(PMDETA)Na(TMP)] to afford **1**. Gibbs energies were calculated at the experimental temperature of 298.15 K and 1 atm, in anisole as solvent. The bottom bar highlights the main reaction steps of the process.*

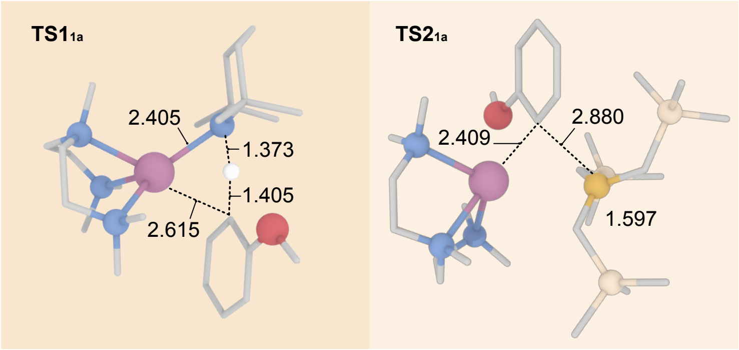

*Figure S2. Optimised transition state structures of **TS11a** and **TS21a**, shown in Figure S1. Relevant bond distances are given in Å.*

Transition State Energies for the Formation of 2 from 1

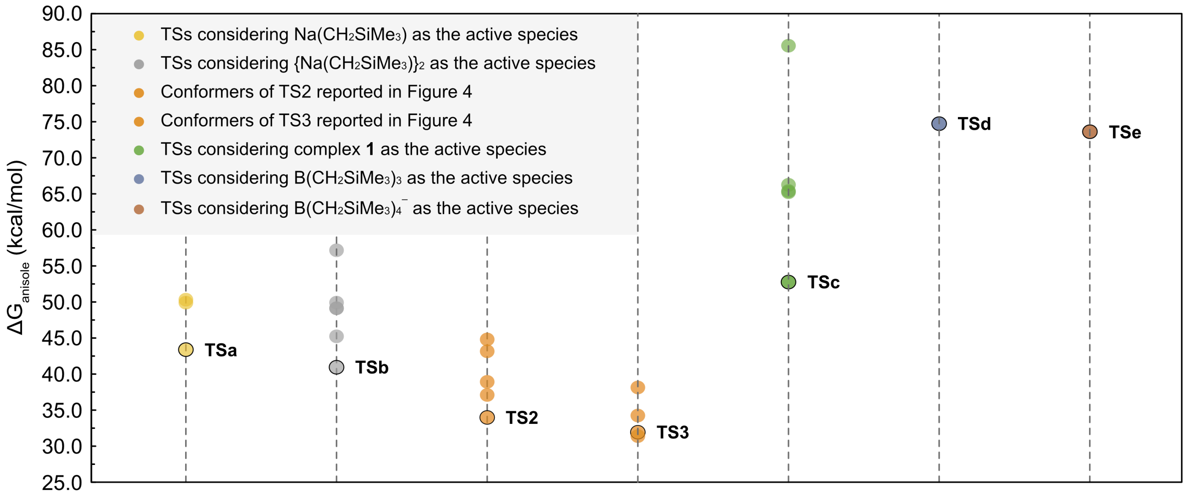

*Figure S3. Gibbs energy barriers computed at 353.15 K and 1 atm for the formation of **2** from the deprotonative borylation of an anisole molecule starting from **1**. All Gibbs energies are referenced to the lowest possible reactive species **1**. The lowest conformers of **TS2** and **TS3** (in orange) are depicted in Figure 4 of the main text.*

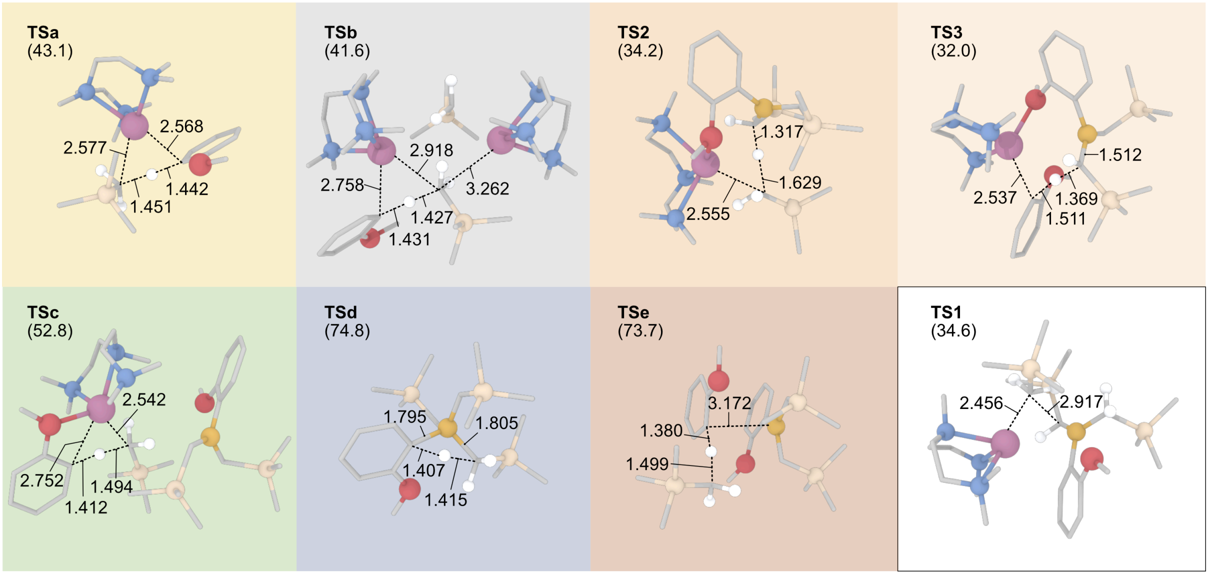

*Figure S4. Lowest energy transition state structures for each of the categories depicted in Figure S4 using the same colour scheme. The transition state structure of **TS1**, mentioned in the main text is also illustrated at the bottom right of the figure with a black frame. Relative Gibbs energies noted in the main text are provided in brackets and the most relevant bond distances are shown in Å.*

Quantum Theory of Atoms in Molecules (QTAIM) Analysis

*Table S3. Selected QTAIM electron density data (ρ(r)) (in a.u.) at the bond critical points (BCP) and relevant bond distances (in Å), showing a multiple bond character for B–C bond in complex **5**. The crystal structure of ^{t}BuB=C(SiMe$_3$)$_2$ has been shown to exhibit a B=C bond.[13]*

	BCP(Na···C)	BCP(B–C)	*r*(B–C)
**Complex**	*ρ*(r)	*ρ*(r)	-
B(CH$_2$SiMe$_3$)$_3$	-	0.178	1.570
^{t}BuB=C(SiMe$_3$)$_2$	-	0.236	1.377
**5**	0.023	0.195 (B=C)<br>*vs* 0.157 (B–Cs)	1.486 (B=C)<br>*vs* 1.611, 1.617 (B–Cs)

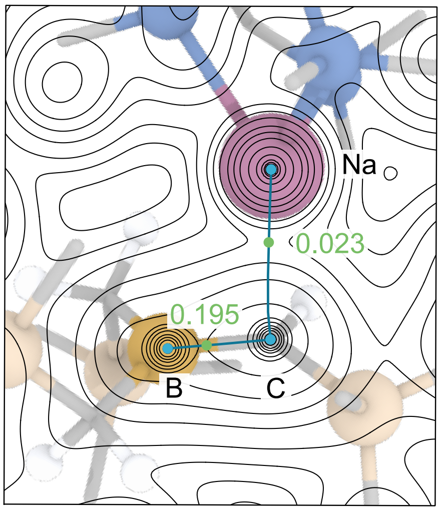

*Figure S5. Electron density contour map for complex **5** generated from the QTAIM analysis depicting the computed bond critical points (BCP) and nuclear critical points (NCP) as green and blue dots, respectively. The ρ(r) values at the BCPs are shown in a.u.*

Mechanism for the hypothetical deprotonative lithiation of $B(CH_2SiMe_3)_3$ with LiTMP·PMDETA

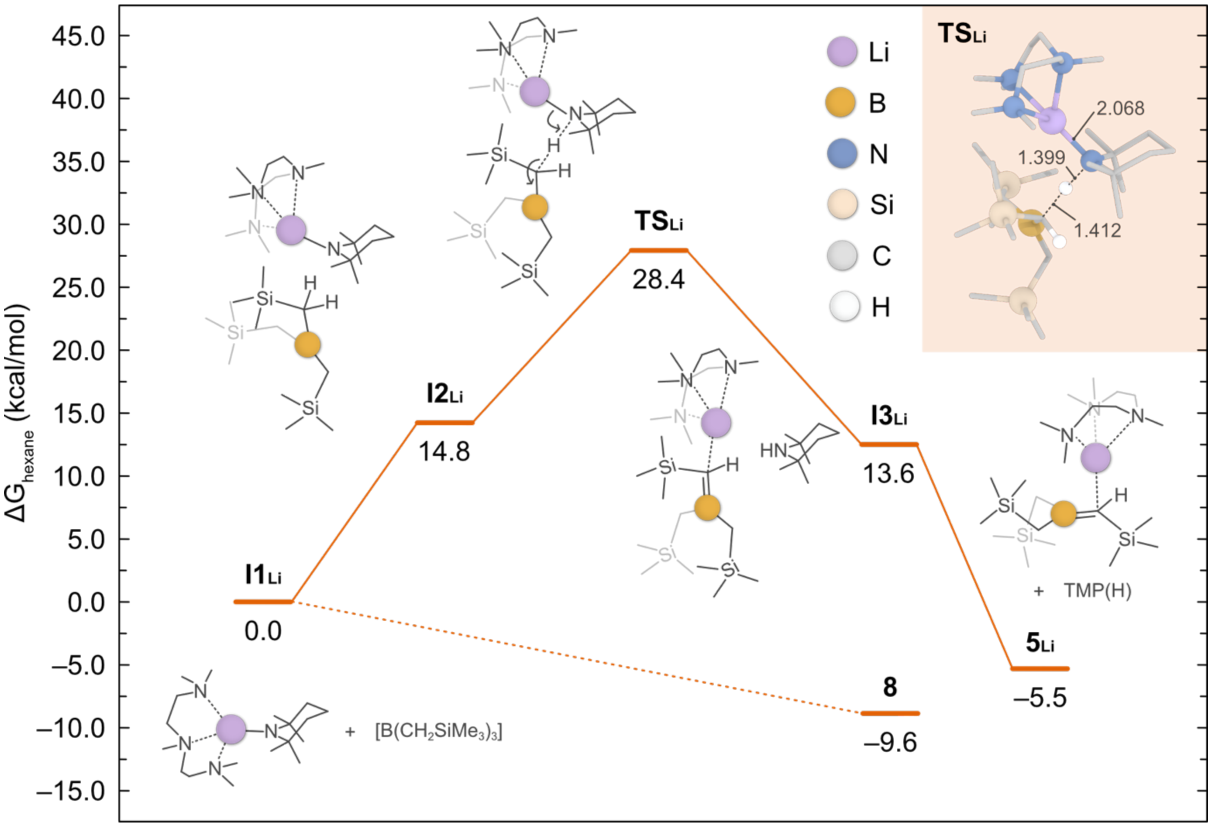

Figure S6. Gibbs energy profile for the deprotonative lithiation of $B(CH_2SiMe_3)_3$ with LiTMP·PMDETA to afford ***5$_{Li}$*** *(the Li analogue of* ***5****), calculated at the experimental conditions of 298.15 K, 1 atm in n-hexane. The detailed mechanism is shown with the aid of curly arrows. In the inset, the* ***TS$_{Li}$*** *is displayed with the relevant bond distances shown in Å. Displacement of the latter leads to* ***I3$_{Li}$****, which does not feature a covalent Li–C bond.*

Cartesian coordinates and energies of the modelled structures

All the DFT data underlying this work, including the Cartesian coordinates and energies of all the modeled structures, is openly accessible via the following ioChem-BD online dataset : https://iochem-bd.bsc.es/browse/review-collection/100/280940/3f1728d4e08ecac8889dd502

References

[1] a) J. C. McMullen, N. E. Miller, *Inorg. Chem.* 1970, **9**, 2291–2295, b) M. U. Kramer, D. Robert, Y. Nakajima, U. Englert, T. P. Spaniol and J. Okuda, *Eur. J. Inorg. Chem.*, 2007, **2007**, 665–674.

[2] Oxford Diffraction (2018). *CrysAlisPro* (Version 1.171.40.37a). Oxford Diffraction Ltd., Yarnton, Oxfordshire, UK.

[3] G. M. Sheldrick, *Acta Cryst*. 2015, **A71**, 3-8.

[4] G. M. Sheldrick, G. M. *Acta Cryst*. 2015, **C71**, 3-8.

[5] O. V. Dolomanov, L. J. Bourhis, R. J. Gildea, J. A. K. Howard, H. Puschmann, *J. Appl. Cryst.* 2009, **42**, 339-341.

[6] J.-D. Chai, M. Head-Gordon, *Phys. Chm. Chem. Phys.* 2008, **10**, 6615–6620.

[7] M. J. Frisch, G. W. Trucks, H. B. Schlegel, G. E. Scuseria, M. A. Robb, J. R. Cheeseman, G. Scalmani,V. Barone, G. A. Petersson, H. Nakatsuji, X. Li, M. Caricato, A. Marenich, J. Bloino, B. G. Janesko, R. Gomperts, B. Mennucci, H. P. Hratchian, J. V. Ortiz, A. F. Izmaylov, J. L. Sonnenberg, D. WilliamsYoung, F. Ding, F. Lipparini, F. Egidi, J. Goings, B. Peng, A. Petrone, T. Henderson, D. Ranasinghe,V. G. Zakrzewski, J. Gao, N. Rega, G. Zheng, W. Liang, M. Hada, M. Ehara, K. Toyota, R. Fukuda, J. Hasegawa, M. Ishida, T. Nakajima, Y. Honda, O. Kitao, H. Nakai, T. Vreven, K. Throssell, J. A. Montgomery, Jr., J. E. Peralta, F. Ogliaro, M. Bearpark, J. J. Heyd, E. Brothers, K. N. Kudin, V. N. Staroverov, T. Keith, R. Kobayashi, J. Normand, K. Raghavachari, A. Rendell, J. C. Burant, S. S. Iyengar, J. Tomasi, M. Cossi, J. M. Millam, M. Klene, C. Adamo, R. Cammi, J. W. Ochterski, R. L. Martin, K. Morokuma, O. Farkas, J. B. Foresman, and D. J. Fox, Gaussian 09, Revision E.01, Gaussian, Inc., Wallingford CT, **2016**.

[8] A. V. Marenich, C. J. Cramer, D. G. Truhlar, *J. Phys. Chem. B* 2009, **113**, 6378–6396.

[9] J. H. Jensen, *Phys. Chem. Chem. Phys.* 2015, **17**, 12441–12451.

[10] R. F. W. Bader, *Chem. Rev.* 1991, **5**, 893–928.

[11] T. Lu, F. Chen, *J. Comput. Chem.* 2012, **33**, 580–592.

[12] E. D. Glendening, J, K. Badenhoop, A. E. Reed, J. E. Carpenter, J. A. Bohmann, C. M. Morales, P. Karafiloglou, C. R. Landis, and F. Weinhold, NBO 7.0., Theoretical Chemistry Institute, University of Wisconsin, Madison, **2018**.

[13] R. Boese, P. Paetzold, A. Tapper, R. Ziembinski, *Z. Anorg. Allg. Chem*. 1989, **1989**, 1057–1060.
